# Mutation in the mouse histone gene *Hist2h3c1* leads to degeneration of the lens vesicle and severe microphthalmia

**DOI:** 10.1016/j.exer.2019.03.024

**Published:** 2019-11

**Authors:** Sharmilee Vetrivel, Natascia Tiso, Andrea Kügler, Martin Irmler, Marion Horsch, Johannes Beckers, Daniela Hladik, Florian Giesert, Valerie Gailus-Durner, Helmut Fuchs, Sibylle Sabrautzki, Martin Hrabě de Angelis, Jochen Graw

**Affiliations:** a Helmholtz Center Munich, German Research Center for Environmental Health, Institute of Developmental Genetics, D-85764 Neuherberg, Germany; b Helmholtz Center Munich, German Research Center for Environmental Health, Institute of Experimental Genetics, D-85764 Neuherberg, Germany; c Helmholtz Center Munich, German Research Center for Environmental Health, Research Unit Comparative Medicine, D-85764 Neuherberg, Germany; d Department of Biology, University of Padova, I-35131 Padova, Italy; e Chair of Experimental Genetics, School of Life Science Weihenstephan, Technische Universität München, D-85354 Freising, Germany; f German Center for Diabetes Research (DZD), D-85764 Neuherberg, Germany

**Keywords:** Histone gene, *Hist2h3c1*, Mutation, Mouse, Eye development, Lens degeneration, Retina hyperproliferation

## Abstract

During an ENU (*N*-ethyl-*N*-nitrosourea) mutagenesis screen, we observed a dominant small-eye mutant mouse with viable homozygotes. A corresponding mutant line was established and referred to as *Aey69* (abnormality of the eye #69). Comprehensive phenotyping of the homozygous *Aey69* mutants in the German Mouse Clinic revealed only a subset of statistically significant alterations between wild types and homozygous mutants. The mutation causes microphthalmia without a lens but with retinal hyperproliferation. Linkage was demonstrated to mouse chromosome 3 between the markers *D3Mit188* and *D3Mit11*. Sequencing revealed a 358 A-> C mutation (Ile120Leu) in the *Hist2h3c1* gene and a 71 T-> C (Val24Ala) mutation in the *Gja8* gene. Detailed analysis of eye development in the homozygous mutant mice documented a perturbed lens development starting from the lens vesicle stage including decreasing expression of crystallins as well as of lens-specific transcription factors like PITX3 and FOXE3. In contrast, we observed an early expression of retinal progenitor cells characterized by several markers including BRN3 (retinal ganglion cells) and OTX2 (cone photoreceptors). The changes in the retina at the early embryonic stages of E11.5-E15.5 happen in parallel with apoptotic processes in the lens at the respective stages. The excessive retinal hyperproliferation is characterized by an increased level of Ki67. The hyperproliferation, however, does not disrupt the differentiation and appearance of the principal retinal cell types at postnatal stages, even if the overgrowing retina covers finally the entire bulbus of the eye. Morpholino-mediated knock-down of the *hist2h3ca1* gene in zebrafish leads to a specific perturbation of lens development. When injected into zebrafish zygotes, only the mutant mouse mRNA leads to severe malformations, ranging from cyclopia to severe microphthalmia. The wild-type *Hist2h3c1* mRNA can rescue the morpholino-induced defects corroborating its specific function in lens development. Based upon these data, it is concluded that the ocular function of the *Hist2h3c1* gene (encoding a canonical H3.2 variant) is conserved throughout evolution. Moreover, the data highlight also the importance of *Hist2h3c1* in the coordinated formation of lens and retina during eye development.

## Highlights

• A dominant small-eye mutant mouse is caused by a mutation in the histone gene *Hist2H3c1.*
• Morpholino-mediated knock-down of *hist2h3ca1* in the zebrafish validated this finding.
• The mutation leads to degeneration of the lens vesicle and retina hyperproliferation.

## 1 Introduction

The ocular system presents an interesting challenge in understanding its development. The vertebrate eye comprises tissues from different embryonic origins: the lens and the cornea derive from the surface ectoderm, while the retina, the epithelial layers of the iris and the ciliary body originate from the anterior neural plate. The timely action of transcription factors and inductive signals ensure the correct development of the different eye components [for a review see Graw (2010)]. On the other side, perturbation of this system can cause isolated or widespread ocular abnormalities including microphthalmia, or even anophthalmia (Plaisancie et al., 2016), that can obstruct the vision at different levels and lead to blindness. At the molecular level, a significant number of genes are involved in the control of eye development. The most notable classes include homeobox genes such as *Lhx2*, *Otx2*, *Pax6*, *Pitx3*, *Rx* and *Six3* (Heavner and Pevny, 2012).

Moreover, chromatin remodelling factors, such as BRG1, have also been found to regulate retinal and lens development (He et al., 2010). More recently, Wolf et al. (2013) demonstrated that loss of CBP and p300, two members of the KAT3 subfamily of histone K-acetyltransferases, leads to a loss of the cell fate determination of the lens, indicating also the importance of core histone modifications for regular lens and eye development. Histone genes are expressed from early development onwards to provide sufficient histones for the rapid cell divisions in early embryogenesis (Graves et al., 1985). The histone genes in higher eukaryotes appear to be arranged as clusters with no apparent order. Most of the histone genes are replication dependent, because new histones are needed during S phase. Correspondingly, their mRNAs are expressed in coordination with DNA replication (Maze et al., 2014). The replication-dependent histone genes in mammals are present in two clusters on separate chromosomes: chromosomes 1 and 6 in humans and chromosomes 3 and 13 in mice (Marzluff et al., 2002). Five genes in histone cluster 1 on mouse chromosome 13 contribute to 65% of H3.2 expression, while the rest is contributed by three genes in the histone gene cluster 2 on chromosome 3 (Wang et al., 1996). *Hist2h3c1* refers to the histone gene cluster 2 at mouse chromosome 3 coding for the first copy (c1) of histone variant H3.2. This gene is present near to the centromeric region (Marzluff et al., 2002).

To further identify novel genes involved in hereditary and congenital eye diseases, we performed a mutagenesis assay using *N*-ethyl-*N-*nitrosourea (ENU) as mutagenic agent (Hrabé de Angelis et al., 2000), and screened the offspring of treated male mice for dominant abnormality of the eye (abbreviation for detected variants: *Aey*, followed by a number). Small-eye mutants are a quite frequent phenotype, and some of them are caused by mutations in the *Pax6* gene (Hill et al., 1991; Graw et al., 2005; Favor et al., 2008, 2009). In contrast to most of the *Pax6* mutants, the small-eye mutant *Aey69* described here is homozygous viable, which makes this mutant line very interesting. Here we describe the molecular characterization of the underlying mutation in the gene coding for a histone H3.2 and the histological and immunohistochemical analysis of the altered process of eye development in the Aey69 mutants. A similar phenotype was obtained in zebrafish embryos using corresponding antisense morpholino oligomers. This new mouse model (*Aey69*) appears as a valuable tool to elucidate the role of histone genes in the complex developmental process of specific organs.

## 2 Materials and methods

### 2.1 Mice

Male C3HeB/FeJ (C3H) mice were treated with ENU (90 mg/kg body weight applied by intraperitoneal injection in three weekly intervals) at the age of 10–12 weeks as previously described (Ehling et al., 1985; Hrabé de Angelis et al., 2000; Aigner et al., 2011) and mated to untreated female C3H mice. The offspring of the ENU-treated mice were screened at the age of 11 weeks for dysmorphological parameters. After the mouse mutant line was established, adult mice were systematically analyzed for their phenotype in the German Mouse Clinic according to standard protocols (Fuchs et al., 2011). Mice were kept under specific pathogen-free conditions at the Helmholtz Center Munich in a 12/12-h dark-light cycle and provided *ad libitum* standard chow (TPT total pathogen free chow #1314; Altromin, Lage, Germany) and water. The use of animals was in accordance with the German Law of Animal Protection, the ARVO Statement for the Use of Animals in Ophthalmic and Vision Research, and the tenets of the Declaration of Helsinki; it was approved by the Government of Upper Bavaria under the registration number 55.2-1-54-2532-126-11.

### 2.2 Eye morphology

To obtain embryos, mice were mated overnight and the presence of a vaginal plug the following morning indicated conception. The noon of that day marked 0.5 days *post coitum*. Pregnant females were sacrificed in a CO_2_ chamber around noon of the respective *post coitum* days to collect the embryos.

For histological analysis, the heads of the embryos were fixed in Davidson's solution overnight, dehydrated in 100% ethanol for 3 times (each for 15 min) and embedded in JB-4 plastic medium (Polysciences Inc., Eppelheim, Germany) according to the manufacturer's protocol. Sectioning was performed with an ultramicrotome (OMU3; Reichert-Jung, Walldorf, Germany). Serial transverse 2-μm sections were cut with a glass knife and stained with methylene blue and basic fuchsin as described previously (Graw et al., 2005).

### 2.3 Linkage analysis

Heterozygous carriers (first generation) were mated to wild-type C57BL/6 J (B6) mice, and the offspring (second generation) were again backcrossed to wild-type B6 mice. DNA was prepared from tail tips of affected offspring of the third generation (G3). For linkage analysis, genotyping of a genome-wide mapping panel consisting of 153 single nucleotide polymorphisms (SNP) was performed using MassExtend, a MALDI-TOF (matrix-assisted laser/desorption ionization, time of flight analyzer) mass spectrometry high-throughput genotyping system supplied by Sequenom [San Diego, CA, USA (Klaften and Hrabé de Angelis, 2005)]. For fine mapping in the critical interval, several microsatellite markers were used.

### 2.4 Sequencing

Exome sequencing was performed by Otogenetics Corporation (Norcross, GA, USA) using DNA of one liver from a homozygous male mutant; bioinformatic analysis of the sequencing data was performed using the cloud analysis platform of DNAnexus (Mountain View, CA, USA). Filtering of the exome-sequencing data was done for the critical interval and for homozygous mutations/polymorphisms predicted leading to an amino-acid exchange as the most likely causative event. As control, we had different mutants of the same genetic background, but with other mutations mapped to different chromosomes.

RNA was isolated from embryonic mouse eyes (E15.5) and reverse transcribed to cDNA using the Ready-to-Go T-primed first strand kit (Invitrogen, Karlsruhe, Germany). Genomic DNA was isolated from tail tips of C3H, B6, CFW, CBA, and 129/SvJ wild-type mice or homozygous/heterozygous embryos (E15.5; on C3H background) according to standard procedures. PCR was performed with a Flex Cycler (Analytik Jena, Jena, Germany) using primers and conditions as listed in Table S1. Products were analyzed by electrophoresis on agarose gels. Sanger sequencing was performed commercially (GATC Biotech, Konstanz, Germany) after direct purification of the PCR products (Nucleospin Extract II, Macherey-Nagel, Düren, Germany). To confirm the mutation in the genomic DNA, the corresponding fragment (in total 463 bp) was amplified from genomic DNA using the primer pair *Aey69-L1* and *Aey69-R1* (Table S1); in the presence of the mutation, a 241-bp subfragment can be digested by the restriction endonuclease *Mnl*I into 2 fragments of 200 bp and 41 bp.

### 2.5 Structural predictions

For structural predictions of missense mutations on the protein structure, we used PolyPhen-2 (http://genetics.bwh.harvard.edu/pph2/) and GOR4 (https://npsa-prabi.ibcp.fr/cgi-bin/secpred_gor4.pl). *In silico* modelling of the mutant and wild-type protein sequences was done using I-Tasser (http://zhanglab.ccmb.med.umich.edu/I-TASSER/; Zhang, 2008). Alignment of the modeled sequences was done using Pymol (The PyMOL Molecular Graphics System, Version 1.2r3pre, Schrödinger, LLC.) and the Root Mean Square Deviation between the aligned structures were calculated.

### 2.6 Transcriptomics

Total RNA was isolated using the RNeasy Midi Kit (Qiagen, Hilden, Germany) and Trizol Reagent (Sigma, Taufkirchen, Germany); only high-quality RNA (RIN>7; RNA integrity number) was used for further analysis. RNA was prepared from whole embryo (E9.5), embryo head (E10.5, E11.5), eye region (E12.5), and eye (E13.5) with n = 4. 300 ng of total RNA were amplified using the Illumina TotalPrep RNA Amplification kit (Thermo Fisher Scientific, Waltham, USA). Amplified cRNA was hybridized to Mouse Ref-8 v2.0 Expression BeadChips (Illumina, San Diego, CA, USA) comprising approximately 25,600 well-annotated RefSeq transcripts and over 19,100 unique genes. Staining and scanning were done according to the Illumina expression protocol. Data was processed using the GenomeStudioV 2011.1 software (gene expression module version 1.9.0) in combination with the MouseRef-8_V2_0_R3_11278551_A.bgx annotation file. The background subtraction option was used and an offset to remove remaining negative expression values was introduced. CARMAweb (Rainer et al., 2006) was performed for quantile normalization. Gene-wise testing for differential expression was done in R (R Development Core Team, 2011) employing the limma *t*-test and Benjamini-Hochberg multiple testing correction (FDR < 10%). To reduce background noise, gene sets were filtered for detection p-values < 0.05 in at least two of three replicates (or at least three of four) in at least one experimental group per comparison. Heatmaps were created in R and pathway analyses were generated by QIAGEN's Ingenuity Pathway Analysis (IPA®, QIAGEN, Hilden, Germany, www.qiagen.com/ingenuity) using Fisher's Exact Test p-values and lens and retina as tissue filters. During the analysis, four samples were excluded, due to quality issues (Ctrl_E9.5_4, Ctrl_E13.5_3) or atypical expression patterns of marker genes for eye development (maybe due to incorrect staging; Aey69_E13.5_3, Ctrl_E12.5_4). Array data have been submitted to the GEO database at NCBI (GSE106941).

### 2.7 Real-time PCR

RNA was extracted using RNeasy mini kit (Qiagen, Hilden, Germany) according to the manufacture's instruction. cDNA was synthesized using Ready-To-Go T-primed first strand kit (Invitrogen) or OmniScript Reverse Transcriptase Kit (Qiagen) or Biozym cDNA synthesis kit including random hexamers (Biozym Scientific GmbH, Oldendorf, Germany). Quantitative real-time PCR was performed with StepOne™ Real-Time PCR System (Applied Biosystem, Darmstadt, Germany). In each reaction, 2 μl cDNA, 0.4 μl reverse and forward primers, 5 μl SYBR Green mix (Bioline, Taunton, USA) and 2.2 μl DEPC-H_2_O were mixed in one well in a 96-well plate and centrifuged briefly. After the initial denaturation step at 95 °C for 15 min, PCR reaction was cycled for 40 times with denaturation at 95 °C for 30 s and annealing-extension temperature at 65 for 30 s. Relative expression was calculated following 2-ΔΔCT method (Livak and Schmittgen, 2001). Primers are listed in Table S1. Statistical analysis was done using REST software and if *p < *0.05, it is reported as statistically different (Pfaffl et al., 2002). The graphs were generated using GraphPad Prism Software version 7 (GraphPad Software Inc., California/USA).

### 2.8 Immunohistochemistry

For immunofluorescent staining, embryos were fixed in 4% PFA overnight and processed for paraffin embedding and sectioned. Embryos were first dehydrated in serial dilution of methanol, followed by bleaching in 3% H_2_O_2_ for 1 h, washed twice in absolute methanol for 10 min each, embedded in paraffin and sectioned at 8 μm by RM 2065*-*microtome (Leica, Wetzlar, Germany).

Embryonic sections were washed in PBS and deparaffinized in Roti-Histol (Roth, Karlsruhe, Germany) followed by rehydration in descending ethanol series. For antigen retrieval in paraffin sections, sections were boiled in 0.01 M sodium citrate buffer (pH 6.4) and cooled slowly by adding MilliQ water. Tissue sections were treated with 1% bovine serum albumin in PBS containing 0.3% Triton X-100, 0.05% Tween-20 (for blocking) and incubated with the primary antibody at 4 °C for overnight. After washing in PBS, sections were incubated with the appropriate secondary antibody for 90 min, counterstained with DAPI and mounted using Aqua-Poly/Mount (Polysciences, Eppelheim, Germany). Images (single plane images and Z-stacks) were obtained with an Olympus confocal microscope (Hamburg, Germany) and analyzed by ImageJ software (https://imagej.nih.gov/ij/). The findings were validated in biological replicates (minimum 2) in a blinded manner. Representative negative controls are shown in the Supplementary Fig. S1. Analysis was not done on areas shown as non-specific stained regions by these images, particularly blood vessels posterior to the lens and disturbed mesodermal cells beneath the RPE and above the cornea. Commercially available and validated antibodies were used and are listed in Table S2.

### 2.9 Statistics

The two-sample *t*-test was used to compare the means of two groups. If the variance was not equal and confirmed by F-test, a nonparametric Mann–Whitney test was used for further statistical analysis. If *p < *0.05, it is reported as statistically different. Regarding the phenotyping analyses at the GMC, tests for genotype effects were made by using Wilcoxon rank sum test, generalized linear models, linear mixed-effects models, *t*-test, Fisher's exact test or ANOVA depending on the assumed distribution of the parameter and the questions addressed to the data. A p-value <0.05 has been used as level of significance; a correction for multiple testing has not been performed. The data of Table S3 was achieved by applying linear models, Wilcoxon rank sum test and Fisher's exact test.

### 2.10 General

If not otherwise mentioned, chemicals and enzymes were from Fermentas (St-Leon-Rot, Germany), Merck (Darmstadt, Germany), or Sigma Chemicals (Deisenhofen, Germany). Oligonucleotides were synthesized by Sigma Genosys (Steinheim, Germany).

### 2.11 Validation in the zebrafish

#### 2.11.1 Zebrafish lines maintenance and handling

Zebrafish embryos and adults were raised, staged and maintained at the Zebrafish Facility of the University of Padova, under standard conditions (Kimmel et al., 1995; Westerfield, 2007). Wild-type lines used in this work included Tübingen, Giotto and Umbria strains (Pauls et al., 2007). The following transgenic lines were used: FGF reporter line *Tg(dusp6:d2EGFP)pt6* (Molina et al., 2007), indicated here as *FGF:EGFP*, TGFβ reporter lines *Tg(12xSBE:EGFP)ia16* (Casari et al., 2014), indicated here as *TGFb:EGFP*, Notch reporter line *Tg(EPV.Tp1-Mmu.Hbb:NLS-mCherry)ia7*, indicated here ad *Notch:mCherry* (Schiavone et al., 2014), *Tg(-5.5ptf1a:DsRed)ia6* and *Tg(ptf1a:EGFP)jh1* (Facchinello et al., 2017), indicated here as *ptf1a:DsRed* and *ptf1a:EGFP*, respectively, *Tg(pax6b:GFP)ulg515*, indicated here as *pax6b:GFP* (Delporte et al., 2008), and *Tg(-2.5neurod1:EGFP)ia50* (Casari et al., 2014), indicated here as *neurod1:EGFP*. All zebrafish experiments were performed in accordance with the European and Italian Legislations, with authorization number 407/2015-PR, obtained from the Ethics Committee of the University of Padua and the Italian Ministry of Health.

#### 2.11.2 Morpholino-mediated gene knock-down

To knock-down the zebrafish *hist2h3ca1* gene, encoding for a protein with 99% identity with mouse Hist2h3c1 (ZFIN ID: ZDB-GENE-030722-8), a translation-blocking morpholino (histMO) oligo, targeting the ATG region, and a control mismatched (mismMO) oligo were designed and synthesized by GeneTools (Table S1). Oligomers were diluted to 100 or 10 μM in 1x Danieau buffer (58 mM NaCl, 0.7 mM KCl, 0.4 mM MgSO_4_, 0.6 mM Ca(NO_3_)_2_, 5 mM Hepes, pH 7.6) plus 1% phenol red. For microinjection experiments, previously mentioned wild-type and transgenic lines were outcrossed with wild-type lines, and 1-cell stage embryos were microinjected with 5 nl of solution. MO-injected embryos (morphants) were raised in egg water with 0.003% PTU (P7629, Sigma Aldrich, Milan, Italy), to reduce pigmentation, and analyzed within 2 days post-fertilization (dpf). Validation of MO specificity was performed by rescue experiments, as described in the next section. Experiments were performed in triplicate, with more than 50 embryos per condition.

#### 2.11.3 Messenger RNA injection experiments

*In vitro* transcription of mouse wild-type (CH3) and mutant (*Aey69*) mRNAs was performed from linearized pCS2 expression clones, using the mMESSAGE mMACHINE Transcription Kit (Ambion, ThermoFisher Scientific; Milan, Italy). For mRNA over-expression and rescue experiments, mRNAs were diluted to 25 or 50 ng/ml concentrations and injected into zebrafish zygotes, either alone or in combination with antisense morpholino oligomers. Experiments were performed in triplicate, with more than 50 embryos per condition.

#### 2.11.4 Whole-mount *in situ* hybridization (WISH)

The following riboprobes were used in WISH experiments: the lens marker *cryba2b* (ZFIN ID: ZDB-GENE-040718-324) and the retinal marker *isl1* (ZFIN ID: ZDB-GENE-980526-112). The *cryba2b* cDNA was obtained from the IRBOp991H0348D clone (Source Bioscience Genome Cube), subcloned into a pCRII TOPO vector (Stratagene, Agilent Technologies, Milan, Italy), linearized with *Kpn*I (Promega, Milan, Italy) and transcribed using DIG- or FLUO-labelling mix and T7 RNA polymerase (Roche, Monza, Italy). The *isl1* probe (Appel et al., 1995; Tokumoto et al., 1995) was transcribed from a pBS KS + clone (insert: +1 to +2265; NM_130,962), linearized with *Xba*I (Promega) and transcribed using DIG- or FLUO-labelling mix and T3 RNA polymerase, (Roche). WISH was performed on zebrafish embryos, previously fixed with 4% PFA/PBS and stored in 100% methanol, following standard protocols (Thisse and Thisse, 2008). For two-colour fluorescent WISH (Lauter et al., 2011), the alkaline phosphatase substrates Fast Red and Fast Blue (Sigma) were used, emitting in the red and far red, respectively. At least 20 embryos per condition were processed in a single tube. For signal comparison, control and treated embryos were co-processed and co-stained in the same tube; controls were recognized by tail tip excision, performed after PFA-fixation and before WISH. All experiments were performed in triplicates.

#### 2.11.5 Microscope imaging of zebrafish samples

After WISH, embryos were post-fixed, mounted in 87% glycerol/PBS and imaged in bright field using a dissecting S8APO microscope (Leica, Milan, Italy) equipped with a Digital Sight DS-L3 camera (Nikon, Florence, Italy). For confocal imaging of Fast Red/Fast Blue fluorescence, embryos were flat-mounted in the same medium and analyzed in a DMI6000 inverted microscope with spectral confocal system SP5 (Leica). Confocal images were processed with Volocity 6.0 software (PerkinElmer). For *in vivo* imaging of fluorescent transgenic lines, embryos were embedded in 2% methylcellulose in PBS with 1x anesthetic Tricaine (0.16 mg/ml) and analyzed with a Leica M165FC dissecting microscope equipped with a DFC7000T camera (Leica). Final figures were assembled using Adobe Photoshop CC (V. 14.0 × 64).

## 3 Results

### 3.1 Generation and phenotyping of the mouse mutant line

**Fig. 1:**
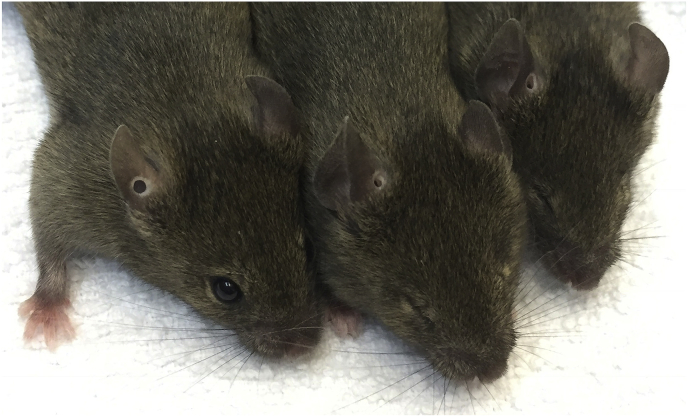
**Small-eye phenotype of *Aey69* mice**. Compared to the wild type (left), the small-eye phenotype is evident in both heterozygous (middle) and homozygous *Aey69* mutants (right) at the age of 8 weeks. This phenotype indicates a dominant mode of inheritance.

Offspring from ENU-treated male mice were screened for different phenotypic parameters including general dysmorphology (Hrabé de Angelis et al., 2000; Fuchs et al., 2012). The mutant *Aey69* (abnormal eyes) was selected because of its small eyes (Fig. 1). When the mutant line was established, it turned out that the homozygous *Aey69* mutants were viable and fully fertile. The standardized phenotyping of this mutant line in the German Mouse Clinic (GMC) revealed only a few altered phenotypes between wild types and the homozygous mutants: increased locomotor activity (hyperactivity) and increased rearing, which was combined with decreased anxiety. An increased body temperature, less body mass and reduced blood lipid values were further characteristics of this mutant line; for details of the various results of the German Mouse Clinic see Table 1, suppl. Table S3 and the mouse phenomap online (www.mouseclinic.de/). Since the microphthalmia was the most severe manifestation of the mutation, we focused in the following experiments on this particular phenotype.

**Table 1:** Results of the German mouse clinic (GMC).

Screens	Test	Phenotype overview of homozygous *Aey69* mouse mutants
**Dysmorphology, Bone and Cartilage**	Morphological observation	Confirmation of microphthalmia
	Bone densitometry	Decreased fat content, increased lean content in females
	X-ray	None
	Click Box	None
**Behavior**	Open Field	Locomotor hyperactivity and increased exploration; signs of decreased anxiety, which may be a secondary confound of the increased activity
	Acoustic startle and Prepulse Inhibition	None
**Neurology**	Modified SHIRPA protocol	Hyperactivity, closed eyes, more tail elevation
	Grip strength	None
	Lactate	None
	Rotarod	Female mutants do not improve compared to controls
**Nociception**	Hot plate assay	None
**Energy Metabolism**	Indirect calorimetry	Body temperature was significantly increased
	Minispec NMR body composition	None
**Clinical Chemistry and Hematology**	Clinical chemistry	*Non-fasted mice* ASAT and LDH activity increased in mutant animals, significantly increased plasma chloride and decreased albumin levels in female mutants; tendency of higher sodium values in mutant mice. *Fasted mice* Statistically significant differences of blood lipid and glucose values in female mutant mice (total cholesterol, HDL-cholesterol and non-HDL-cholesterol); Triglyceride values were significantly decreased in mutant females; glycerol levels slightly decreased in both male and female mutants
	Hematology	None
	IpGTT	Slightly increased fasted glucose level
**Immunology**	Flow cytometry	Subtle alterations in T cell subsets in females
	Immunoglobulin levels	Decrease in the levels of IgG1 and IgM
**Allergy**	IgE level	None
**Steroid Metabolism**	DHEA level	Slightly increased in male mutants
	Testosterone level	None
**Cardiovascular**	Non-invasive tail-cuff blood pressure measurement	None
	Heart weight	None
**Lung Function**	Whole body plethysmography	Only body mass related differences between female groups
**Pathology**	Macroscopic analysis	Confirmation of anophthalmia
	Histology	None

The Eye Screen was removed from the list, because the characterization of the eye development is the objective of this paper. Moreover, because of the microphthalmic/anophthalmic phenotype and the severe ocular malformations, our routine test systems could not be applied.

p-values are given in Supplementary Table S3; all data will be available through the mouse PhenoMap online (www.mouseclinic.de/).

### 3.2 Histological analysis of the microphthalmia phenotype

**Fig. 2:**
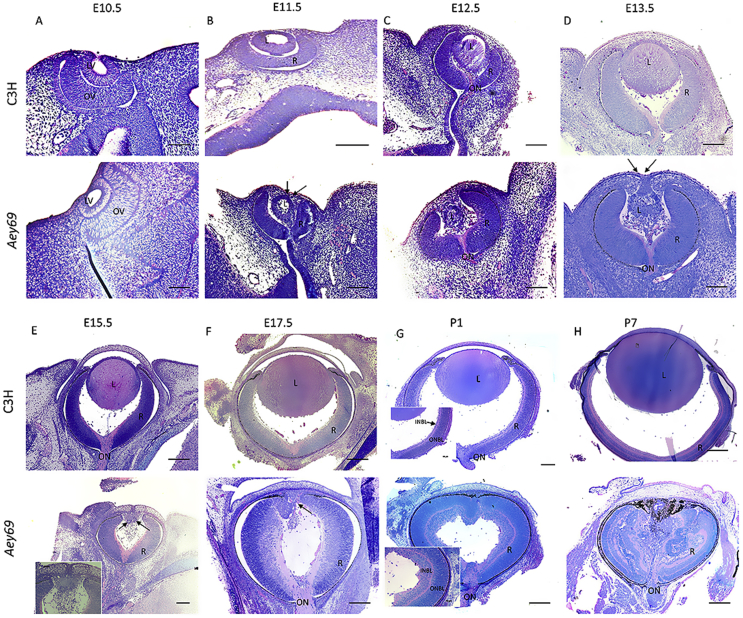
**Histological analysis of the microphthalmia phenotype.** The comparative histological staining between the wild-type and homozygous *Aey69* eyes are shown. Eye development is demonstrated from embryonic day E10.5 until postnatal day (P) 7. The figure summarizes the major disruptions in development starting from the lens vesicle stage of E11.5 (a–d) and the overgrowing of the retina into the empty lens space after birth (e–h). In particular, at E11.5 in the wild type there is no surface ectoderm connection between the future cornea and lens. However, in the mutant the surface ectoderm connection is maintained (as highlighted by black arrows) through the embryonic stages of E11.5-E13.5, when the lens gradually disappears. Further changes in later embryonic stages are also highlighted by their respective black arrows: at E15.5 increased infiltration of periocular mesenchymal cells into the mutant vitreal space, at E17.5 altered bending of retinal layers anterior to the cornea, and at P1 the mutant retinal layers are observed to be much thicker compared to the wild type. The bars indicate 100 μm at E10.5-E12.5, 50 μm at E13.5 - E15.5, and 0.1 mm at P7. L, lens; R, retina; ON, optic nerve; INBL, inner neuroblastic layer; ONBL, outer neuroblastic layer.

Histological analyses of eye development in the homozygous *Aey69* mutant mouse are demonstrated in Fig. 2. In initial experiments, we compared histological data between all three genotypes; however, since the features of the heterozygous and the homozygous *Aey69* mutant were without obvious differences (Supplement Fig. S2 for E13.5), we focused on the comparisons between wild-type and homozygous mutant mice. The formation of the lens vesicle at E10.5 in the mutant was not markedly different from the wild type (Fig. 2a). However, at E11.5 and E12.5 the shape of the mutant lens vesicle appeared smaller and disorganized (Fig. 2b and c). Subsequently, at E13.5 it became obvious that the transient connection between the surface ectoderm (the future cornea) and the lens vesicle was not detached. Moreover, the mutant lens was not filled by well-organized primary lens fiber cells (as it was in the wild-type lens), but instead by pycnotic and disorganized cells (Fig. 2d). At these stages, the retina did not seem to be affected in the histological sections. However, starting from the embryonic stage of E15.5, changes in the cornea and retina were observed (Fig. 2e–h). At E15.5, the cornea seemed to be much thicker in the mutant as compared to the wild type, and there was still a remnant lens stalk that failed to separate from the cornea. Increased infiltration of periocular mesenchymal cells into the vitreal space was also observed (Fig. 2e). At E17.5, the retina was observed to be much thicker and larger in the mutant as compared to the wild-type; also, aberrant bending of retinal layers anterior to the cornea was observed (Fig. 2f). At P1, the retinal pigmented epithelium (RPE) did not stop at the tips of the retina like in the wild types, but covers the entire anterior part of the eye. This abnormal growth of the RPE did not affect the establishment of outer and inner neuroblastic layers in the neural retina (see inset in Fig. 2g). Finally, after birth, RPE and other layers of the retina invaded massively the central part of the eye, filling up the space usually occupied by lens and vitreous (Fig. 2h).

From these histological features, several questions arose:

- What is the underlying mutation in *Aey69* leading to this severe ocular phenotype?
- What cellular processes are disrupted in the lens vesicle of the *Aey69* mutants?
- Do all retinal cell types contribute to the retinal overgrowth?

These questions will be addressed in the following sections.

### 3.3 Mapping and sequencing of the underlying mutation

**Fig. 3:**
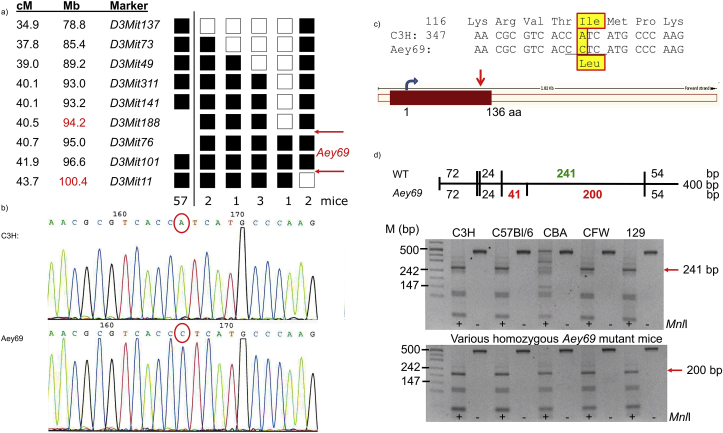
**Linkage and sequence analysis of *Aey69* mutation.** a) Haplotype analysis defines the critical interval between the markers *D3Mit188* and *D3Mit11* at mouse chromosome 3. The analysis was performed in two steps separated by the black line; the markers *D3Mit188* and *D3Mit76* were used only in the 9 mice with a recombination between *D3Mit141* and *D3Mit11*. The numbers of mice for each haplotype are given; 7 mice of the F2 panel had the B6 allele of all markers tested, but carried the *Aey69* mutation. Black squares are heterozygotes, and empty squares represent homozygotes for the C57Bl/6 J allele. The red arrows mark the critical interval for the underlying mutation; the genetic distances (given in cM) and the exact physical positions of the markers (given in Mb) are from the MGI database (http://www.informatics.jax.org/; Dec. 2018). b) Sanger sequencing confirmed the exome sequencing data (c. 358 A-> C; red circles). c) The change in the amino acid sequence (Ile120Leu) is given below and boxed in yellow with a red surrounding line; the underlined DNA sequence (CCTC) demonstrates the new *Mnl*I restriction site in the mutants. Schematic drawing of the mouse *Hist2h3c1* gene (ENSEMBL) is given below the nucleotide sequence; the red arrow points to the site of the mutation at the C-terminal end of this single-exon gene. d) The novel *Mnl*I restriction site is present in all homozygous mutant mice tested. It is absent in 5 tested wild-type strains indicating that it is a mutation and no widespread polymorphism. The schema above the gels explains the digestion pattern of the fragment, and the size of the critical bands is given in red or green. The red arrows point to these critical bands and their sizes are indicated; +, with *Mnl*I restriction enzyme; -, without restriction enzyme.

In a genome-wide linkage analysis using SNP markers, the mutation was mapped to chromosome 3. Fine mapping using microsatellite markers defined a critical interval between *D3Mit188* and *D3Mit11*; the markers *D3Mit76* and *D3Mit101* did not show any recombination among 80 G3-mice tested (Fig. 3a). Based upon this positional information, we tested several candidate genes (*Cef3, Cgn, Gja8, Pogz, Selenbp* and *Selenbp2*). Among them, only a mutation in the *Gja8* gene (coding for connexin50) was observed (c.71 T→C; Val24Ala). Since all mutations reported in the *Gja8* gene in the mouse (and in its human homologue *GJA8*) led to lens opacities (cataracts) (Graw et al., 2001; Schmidt et al., 2008; Xia et al., 2012; Berthoud and Ngezahayo, 2017), but never to microphthalmia without a lens (as observed in the adult heterozygous *Aey69* mutant mice), we excluded *Gja8* as a candidate gene for *Aey69*.

Exome sequencing detected the *Aey69* mutation in the *Hist2h3c1* gene at c.358 A > C resulting in an Ile- > Leu exchange at amino-acid position 120 (Ile120Leu). The mutation was confirmed using classical Sanger sequencing (Fig. 3b and c) and by restriction digest using *Mnl*I, which did not cut the mutated sequence, but not the wild-type fragment (Fig. 3d). The PCR fragment remained intact in five tested wild-type mice of different genetic background, but was digested in all 5 *Aey69* mice from our breeding colony. Therefore, we concluded that this missense mutation is a true mutation and not a polymorphism; this interpretation is also supported by the ENSEMBL database; there is no polymorphic site upstream of nt 402; the polymorphic region affects just the last 3 C-terminal amino acids (ENSMUST00000176059.1).

Because of the unexpected finding of a mutation in a histone gene causing microphthalmia without a lens, we tried to separate the two candidate genes *Gja8* and *Hist2H3c1* genetically. Unfortunately, they were so close together (*Gja8* at 96.9 Mb and *Hist2H3c1* at 96.2 Mb; ENSEMBL release 94 - October 2018) that we stopped after 5 generations outcrossing without a positive result. This outcome was underlined by the haplotype analysis demonstrating that the two markers *D3Mit76* (95.0 Mb) and *D3Mit101* (96.6 Mb) did not show a recombination with the *Aey69* mutation (Fig. 3a). Moreover, it can be noticed that in all our out- and back-cross breeding no difference of the microphthalmia phenotype between C3H and C57BL6 mice was observed.

**Fig. 4:**
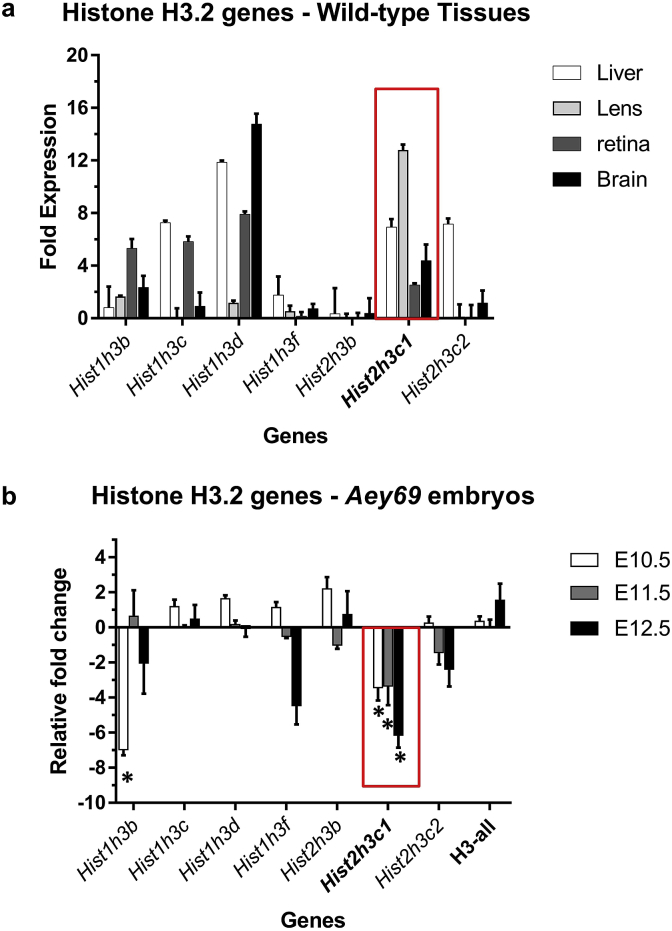
**QPCR analysis of Histone H3.2 coding genes.** a) Relative expression levels of histone genes in the wild-type tissues of brain, liver, lens and retina. *Rplp0* (ribosomal protein, large, P0) was taken as the housekeeping gene, and analysis was done using the relative expression method. Values are given as fold expression levels ± SEM; n = 3 for each tissue type. The gene of interest, *Hist2h3c1*, is highlighted by a red box; *Hist2h3c1* was found to be the most highly expressed H3.2 encoding gene in the lens. b) Gene expression changes in the embryonic tissues of *Aey69* at the embryonic stages of E10.5-E12.5 using the -2ΔΔct method; the respective wild-type tissues were used as the control, and *Rplp0* was taken as the housekeeping gene. Values are given as fold expression levels ± SEM. n = 3 for each embryonic stage. Statistically significant differences of the expression levels (p < 0.005) are marked by an asterisk. The mutated gene *Hist2h3c1* (red box) was found to be significantly downregulated through these stages.

Based upon the MGI database, *Hist2h3c1* is expressed in the retina, but also in liver and spleen. It is one of the eight genes in the mouse histone gene clusters encoding for the protein histone H3.2. To test for any tissue specific dependence amongst the histone clusters expression pattern of these genes were analyzed in three wild-type tissues – brain, retina, lens and liver (Fig. 4a). Since no specific primers could be designed for the *Hist2h3e* gene, the analysis was performed on the remaining 7 histone genes only. Among the H3.2 encoding genes, *Hist2h3c1* was found to be most highly expressed gene in the lens (fold expression level >5; compared to the housekeeping gene *Rplp0*). With regard to embryonic stages of the *Aey69* mutants (Fig. 4b), we observed a significant downregulation of the histone gene *Hist1h3b* in E10.5, and downregulation of *Hist2h3c1* through the stages E10.5-E12.5 (p < 0.05), but the overall expression levels of H3 genes was not dramatically changed (using universal H3primers; Banday et al., 2014).

### 3.4 Structural prediction of the mutant Hist2h3c1 protein

*Hist2h3c1* (http://www.informatics.jax.org/marker/key/86142) is one of the eight single-exon histone genes and encodes for a histone H3.2 variant (https://www.uniprot.org/uniprot/P84228). The prediction of the consequences of this mutation by PolyPhen-2 was “possibly damaging” with a score of 0.726 (sensitivity 0.78 and specificity 0.85). GOR4, a secondary protein structure prediction program, suggests a shortened part of the coiled-coil domain (9 amino acids in the wild type to just 6 amino acids in the mutant protein) corresponding to an extension of the flanking α-helical regions (from 5 to 6 amino acids forming a N-terminal helical domain, and from 9 to 11 amino acids forming a C-terminal helical domain). I-Tasser predicted the wild-type and mutant proteins to be structurally distinct by a RMSD (Root-Mean-Square Deviation) value of 1.352 Å.

### 3.5 Differential analysis of transcripts

**Fig. 5:**
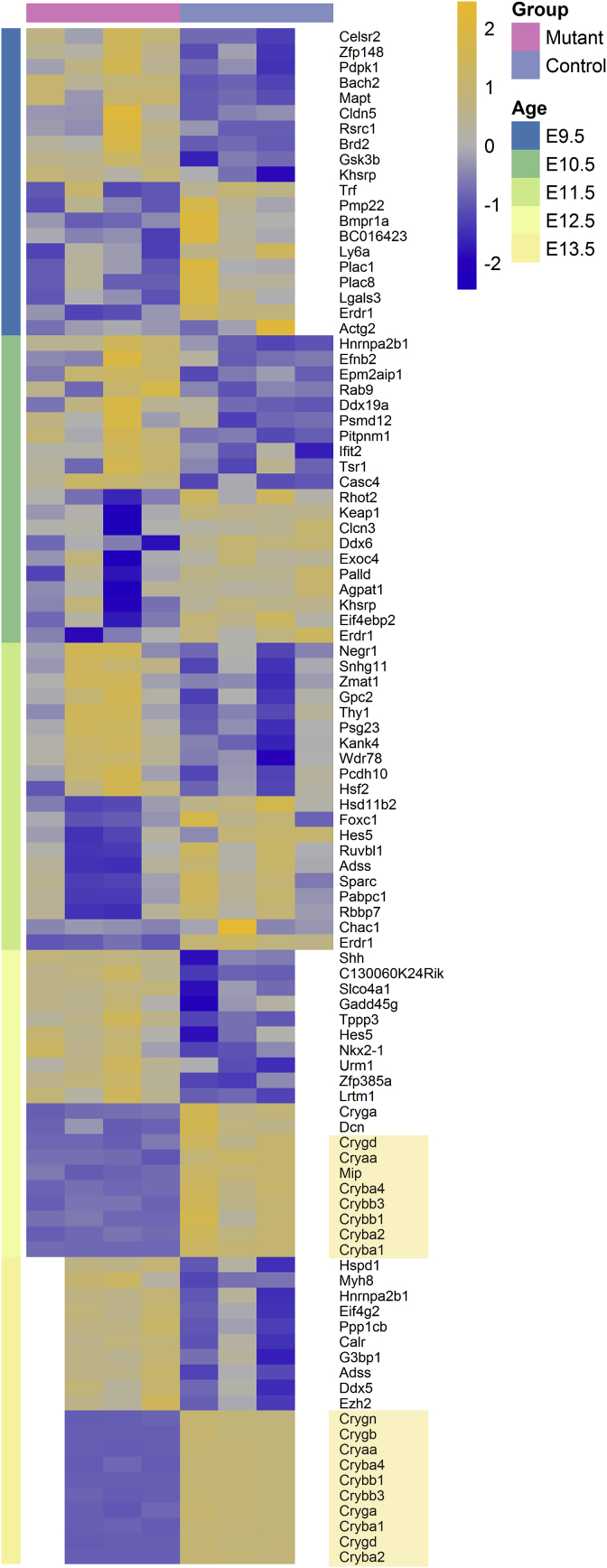
**Microarray analysis of differentially regulated genes in *Aey69* embryos.** Heatmap of the top analysis-ready genes from our Ingenuity analysis, regulated between *Hist2h3c1* mutant embryos and controls. Genes were ordered by fold-change within each stage and relative gene expression values are shown across samples (z-scales to mean expression per row). The downregulated crystallin genes (and *Mip*) are highlighted in beige.

For a better understanding of the changes during early eye development, we performed a microarray analysis of transcriptomic changes in *Hist2h3c1* mutant embryos and their wild-type littermates using whole embryos (E9.5), embryo heads (E10.5, E11.5), tissues of the eye region (E12.5), and whole eyes (E13.5). We defined sets of regulated genes with p < 0.05 (limma *t*-test p-value) and applied additional filters for fold-change and background reduction as described in the methods section. This approach resulted in 376 regulated genes at E9.5, 157 genes at E10.5, 420 genes at E11.5, 847 genes at E12.5, and 739 genes at E13.5. These gene sets were further studied using the Ingenuity Pathway Analysis software, and the top analysis-ready genes for each stage are shown in Fig. 5. These results clearly indicated that lens-specific genes like αA-, β- and γ-crystallins, as well as *Mip,* are downregulated in the mutant eye at E12.5 and E13.5. Similarly, *Gja8* is downregulated at E12.5 (−1.4x, p < 0.05) and E13.5 (−2.7x, FDR<10%), but its expression is low and therefore, it did not pass the detection p-value filter (and is not included into Fig. 5). Taken together, these data indicated that at these stages the lens vesicle did not develop properly to a lens. Surprisingly, none of the key transcription factors of eye development (*Pax6*, *Otx2*, *Sox2*) were found to be among the top-altered genes in the early stages except *Bmpr1a*, encoding a receptor for BMPs, of which BMP4 and BMP7 are known to be important for early eye development (for a recent review see Williamson and FitzPatrick, 2014). Moreover, pathway analysis revealed as the top-altered pathway integrin-linked kinase (ILK) signaling (Supplementary Fig. S3), which was demonstrated being required for lens epithelial cell survival, proliferation and differentiation (Teo et al., 2014). To make sure that lens cell differentiation and survival was affected in the *Aey69* mutant, we focused in the next step on the characterization of the lens vesicle disappearance including the validation of the loss of the lens-specific genes.

### 3.6 Disappearing lens vesicle in *Aey69* mutants

**Fig. 6:**
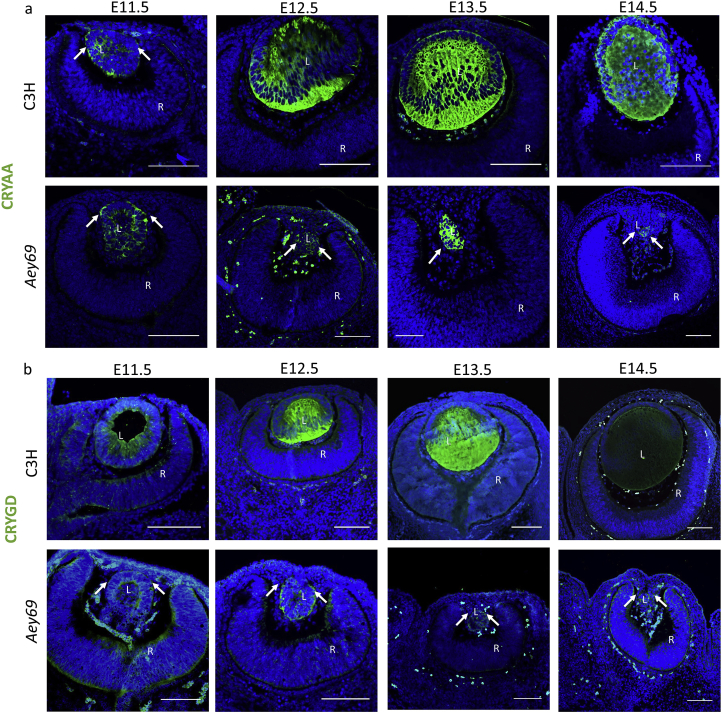
**Lens development in *Aey69* mutants.** The lens-specific marker CRYAA (a) and CRYGD (b) were used to characterize the early lens from the stages of E11.5-E14.5. At E11.5, no major change was observed in the distribution of crystallins between the wild type and mutant lens (marked by their respective arrows). However, through the stages of E12.5 – E14.5 the arrows highlight clearly the decreased CRYAA and CRYGD expression and a diminishing lens region in the mutant. The bars indicate 100 μm; n = 3 for each embryonic stage; L, lens; R, retina; ON, optic nerve.

To understand, whether there is any kind of lens material in the mutant eyes, we checked by immunohistochemistry for the presence (or absence) of markers like CRYAA (Fig. 6a) and CRYGD (Fig. 6b); these proteins are considered to be expressed in lens fiber cells, but not in other ocular cells (Graw, 2009). The lens specific expression of CRYAA and CRYGD indicated clearly that there was lens material expressing these proteins in the mutant, but their expression pattern was not comparable to the wild type. There was an obvious decrease of lens-specific proteins in the developing mutant eyes from E12.5 onward. It can be concluded that the failed separation of the surface ectoderm does not prevent the expression of lens specific proteins, but rather stopped these lens cells from successfully differentiating into lens fiber cells.

**Fig. 7:**
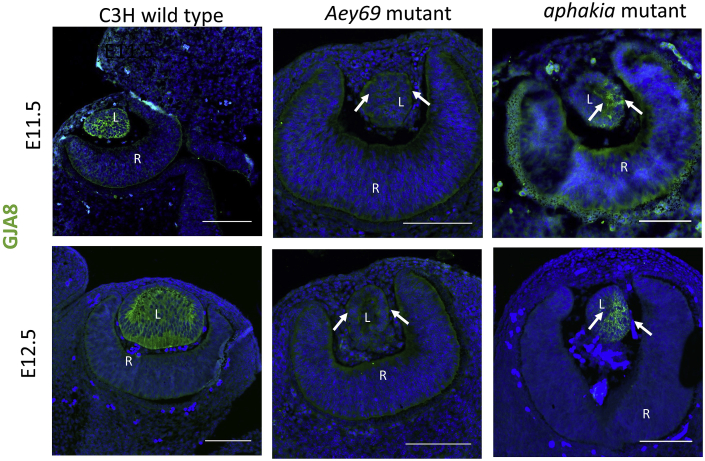
**GJA8 in early eye development.** The immunohistochemical distribution of GJA8 is shown at E11.5-E12.5 in wild type, *Aey69* mutant and similar microphthalmic mouse model *aphakia*. The shrinking lens region is marked in the mutant models by white arrows. No obvious immunohistochemical localization of Gja8 in the mutant eyes at the stages of E11.5-E12.5 was observed. The bars indicate 100 μm; n = 3 for each embryonic stage; L, lens; R, retina; ON, optic nerve.

The cataractous role of *Gja8* mutations has been well documented from post-natal stages; however, early embryonic expression patterns of *Gja8* have still not yet been defined. Since it was proposed by deRosa et al. (2007) that *Gja8*-encoded Cx50 might be involved in primary fiber cell elongation, we analyzed the expression pattern of *Gja8* during early embryonic stages (E10.5-E12.5). Due to the similarity of the lens pathology of the *Aey69* mutants with the *Pitx3* mutant mouse *aphakia* (*ak*) (Semina et al., 2000; Ahmad et al., 2013), we also tested for the immunohistochemical distribution of GJA8 in the *aphakia* mouse (the absence of *Cryaa* transcripts in the developing lens was reported earlier by Grimm et al., 1998). With regard to the localization, comparative immunohistochemical analysis of GJA8 expression was performed in wild type, *Aey69* and *aphakia* mutants at E11.5 and E12.5 (Fig. 7). At E11.5, in the wild type, GJA8 expression covers the entire lens vesicle, while in *aphakia* mutant the expression seemed to be highly irregular and restricted to one part of the mutant lens vesicle. Interestingly, at this stage, no GJA8 expression was observed in the *Aey69* mutant. Furthermore, at E12.5, GJA8 expression became more restricted to the region beneath the future lens epithelial layer. In the *aphakia* mutant, the one sided expression of GJA8 continued in the disorganized lens structure. In the *Aey69* mutant, however, no comparable expression to either the wild type or *aphakia* mutant was found at both stages. In fact, no characteristic expression of GJA8 can be observed in the *Aey69* mutant.

**Fig. 8:**
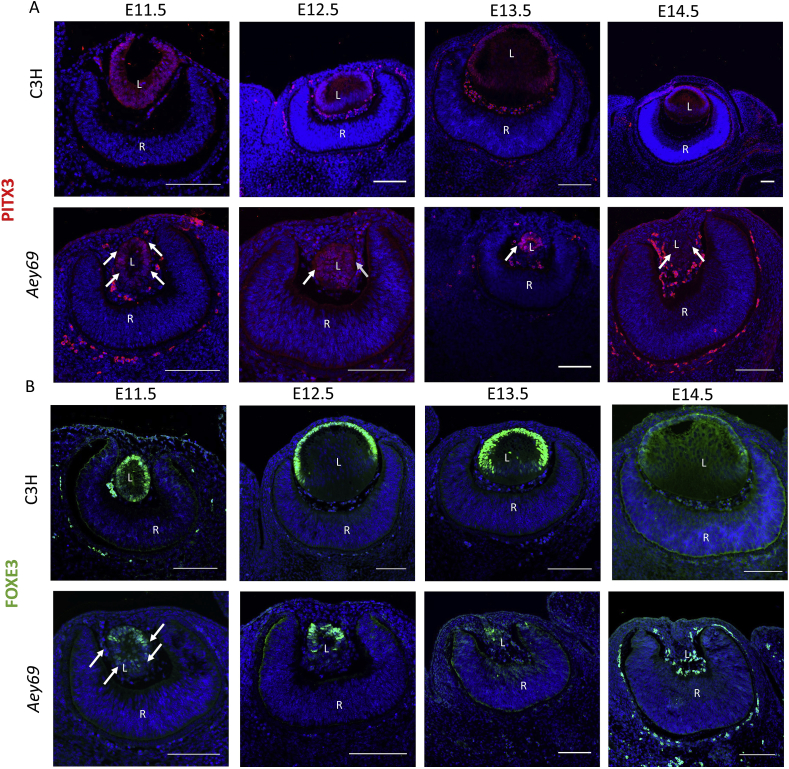
**Changes in the expression patterns of the transcription factors PITX3 and FOXE3 in the *Aey69* mutant lens.** a) The transcription factor PITX3 was used to characterize the alteration in lens development from E11.5 -E14.5, when the lens structure diminishes. Similar to CRYAA, for PITX3 at E11.5 there was no major change in the distribution in the wild type and mutant lens (marked by their respective white arrows) and through the stages of E12.5 – E14.5 the arrows highlight clearly the diminishing PITX3 expression in the shrinking mutant lens. The bars indicate 100 μm (n = 3 for each embryonic stage). L, lens; R, retina; ON, optic nerve. b) The lens-specific transcription factor FOXE3 was used to identify any disruptions in lens development starting from E11.5. The arrows marking the mutant lens at E11.5 clearly indicate reduced expression of FOXE3 at E11.5. The bars indicate 50 μm; n = 3 for each embryonic stage; L, lens; R, retina; ON, optic nerve.

Since a mutation in the *Pitx3* gene is causative for the absence of the lens vesicle in the *aphakia* mutant, we checked its expression in the Aey69 mutant. Interestingly, PITX3 lens expression was maintained in the wild-type and mutant lens vesicle at E11.5. In the subsequent stages, PITX3 expression became limited to the future lens epithelium in the wild type, however no such restriction was found in the mutant, and PITX3 seemed to be distributed all over the lens area. Later, a decrease in PITX3 stained area was observed from E12.5-E14.5 (Fig. 8a). The decreasing pattern of PITX3 follows the trend of the crystallin expression pattern indicating a dying lens structure wherein the lens markers are gradually lost. Since *Foxe3* is a direct target of PITX3 (Ahmad et al., 2013), and since the *Foxe3* mutant mice *dyl* (dysgenetic lens; Blixt et al., 2000) did not show proper lens development, we also checked for the presence or absence of FOXE3 in the *Aey69* mutants (Fig. 8b). Consistent with our other findings, we also observed that FOXE3 rapidly disappeared in the *Aey69* embryonic eye (Fig. 8b). The distribution was not uniform over the mutant lens vesicle in comparison to the wild type. Comparing the images in Fig. 8, the decrease of the PITX3 expression in the mutant lens is not as strong as the decrease of the FOXE3 expression.

**Fig. 9:**
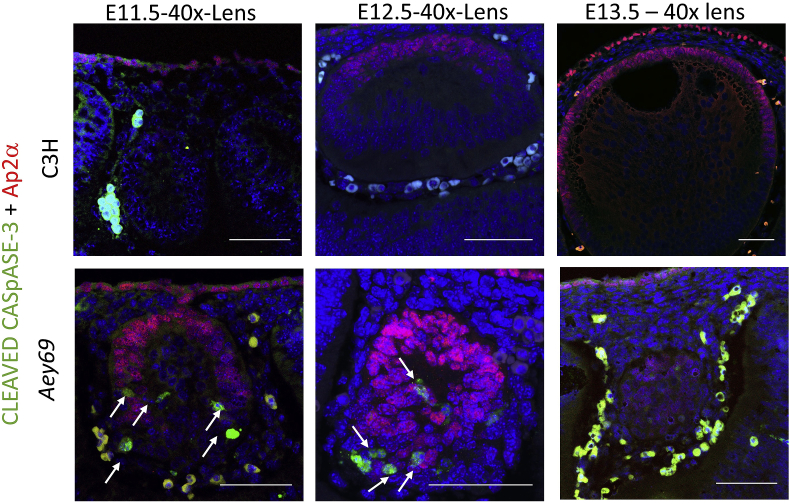
**Disappearing lens vesicle and Ap2α in lens and retina.** The apoptotic marker Cleaved Caspase 3 (green) was used to characterize apoptotic events during ocular development from E11.5 -E13.5, when the lens structure diminishes. The arrows marking the mutant lens at E11.5-E12.5 clearly indicate that the apoptotic death of the lens structure. The ocular transcription factor Ap2α (red) was used to characterize transcriptional regulation of ocular development from E11.5 -E13.5, when the lens structure diminishes. The apoptotic process leads to a shrinking lens as it can be observed from the decreased number of Ap2α-positive cells in the subsequent stages. The bars indicate 50 μm.

The rapid degeneration of the lens between E11.5 and E13.5 with numerous pycnoytic nuclei and apparent failure of fiber cell differentiation (Fig. 2), led us to examine if lens vesicle cells were undergoing apoptosis. Double labelling with AP2α, a lens epithelial marker, and cleaved caspase-3, indicated that only posterior vesicle cells, which were only weakly or not labelled with AP2α, were undergoing apoptosis (Fig. 9). These results suggest that early differentiating fiber cells were undergoing apoptosis.

### 3.7 Retinal hyperproliferation and overgrowth

**Fig. 10:**
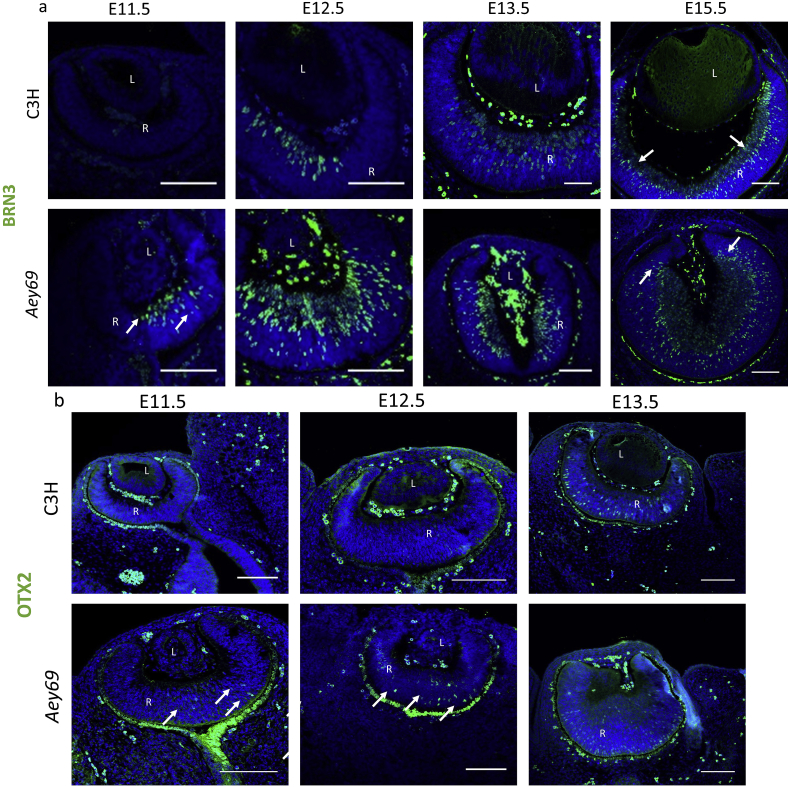
**Retinal development in *Aey69* mutants.** a) The ganglion cell specific marker BRN3 was used to characterize the early retina developmental changes and associated hyperproliferative events. The arrows in the mutant retina at E11.5 clearly indicate an early overexpression of BRN3-positive retinal cells. This overexpression does not affect the expansion of the BRN3 positive cells to the prospective ganglion cell layer in mutant retina at E15.5 similar to the wild type (marked by arrows in the respective sections). b) OTX2 was used to characterize the early changes in *Aey69* mutant retina at the stages of E11-5-E13.5. The results indicate an early appearance of OTX2-positive retinal cells in the mutant at E11.5 and E12.5 (indicated by white arrows at the respective stage). n = 3, for each embryonic stage; bars indicate 100 μm; L, lens; R, retina; ON, optic nerve.

The retina comprises seven primary cell types: rod and cone photoreceptors, amacrine cells, retinal ganglion cells (RGCs), horizontal cells, bipolar cells and Müller glia. These cells are formed from a common pool of retinal progenitor cells during development in a characteristic, but overlapping, order (Livesey and Cepko, 2001). Amongst the different cell types, BRN3-positve retinal ganglion cells and OTX2-positive cones represent the earliest retinal progenitor population starting around E11.5 and E12.5 respectively (Brzezinski et al., 2010; Pan et al., 2005; Rodgers et al., 2016). Therefore, these two markers were used to characterize the early retinal development in the wild type and Aey69 mutant from E11.5 onwards (Fig. 10). Immunostaining showed the foremost BRN3 expression in the central retina at E12.5 of the wild type. As retinal development progresses, the expansion of BRN3-positive population was seen around the peripheral retinal regions (E13.5) and extends to the migrating retinal ganglion cells (RGC) to form the prospective ganglion cell layer (GCL) of the developing retina. However, in the mutant retina the foremost expression of BRN3 and OTX2 started from E11.5, and comparatively more BRN3- and OTX2-positive cells were observed through the stages of E12.5-E13.5.

**Fig. 11:**
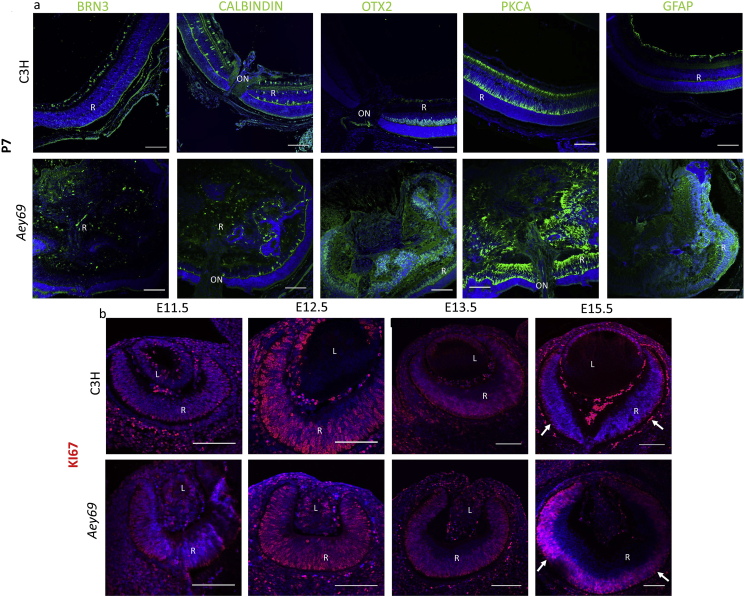
**Hyperproliferation in the *Aey69* mutant eye.** a) Antibodies labeling diverse retinal cell types were used to characterize the retina at P7. The wild-type images clearly indicate that at P7 there is a stratified retina with distinct cell types: Calbindin-positive horizontal and amacrine cells, PKCα-positive bipolar cells, OTX2-positive photoreceptors and bipolar cells, GFAP-positive Müller cells, and BRN3-positive retinal ganglion cells. In the *Aey69*, these cell types were present covering the entire ‘empty lens area’ of the mutant eye. The bars indicate 50 μm; n = 3 for each embryonic stage; L, lens; R, retina; ON, optic nerve. b) KI67 immunostaining was used to characterize proliferation in the developing eye of wild types and mutants. The results indicate differences in the distribution of KI67 between the wild-type and mutant eyes. At E15.5, in the wild-type retina KI67 positive cell population seems to be restricted to the future outer neuroblastic layer (marked by arrows). However, in the mutant the arrows indicate that the region occupied by the Ki67-stained cells is comparatively larger. The bars indicate 100 μm; n = 3 for each embryonic stage. L, lens; R, retina; ON, optic nerve.

At P7, most retinal cells occupied their final positions within the retina. To see, whether all major retinal cell types contribute to this overgrowth in the *Aey69* mutants, the localization of the major retinal cell types was assessed using Calbindin (horizontal, amacrine and ganglion cells), Protein kinase Cα (bipolar cells), OTX2 (photoreceptors and bipolar cells), GFAP (Müller cells) and BRN3 (RGC). The main conclusion from this analysis is that all major retina layers are present in the mutant mice, but their retinal architecture is lost: the whole retina appeared as a collapsed structure, and the space was filled by cells with retinal characteristics (Fig. 11a).

To know whether increased proliferation is causing the over-expression of retinal population, immunohistochemical distribution of pan cell cycle marker Ki67 was done (Fig. 11b). Ki67 was found to label completely the entire ocular section from E11.5-E13.5 in a similar manner in both the wild type and mutant. At E15.5, in the wild type retina Ki67 stained cells were restricted to a single layer posterior to the RPE. However, in the mutant the region occupied by the Ki67 stained cells was comparatively larger (it should be noticed that there was some non-specific staining at the blood vessels and at the mesodermal cells as obvious from the comparison to the negative control section, Fig. S1). Taken together, increased expression of retinal progenitor cells at early embryonic stages (E11.5-E12.5) were followed by increased proliferative activities in the retina at E15.5. This overdrive of retinal proliferation events could be hypothesized to be the spear head of the retinal overgrowth covering the entire eye at the postnatal stages (Fig. 2e–h).

### 3.8 Confirmation of *Hist2h3c1*-induced microphthalmia in zebrafish eye development

**Fig. 12:**
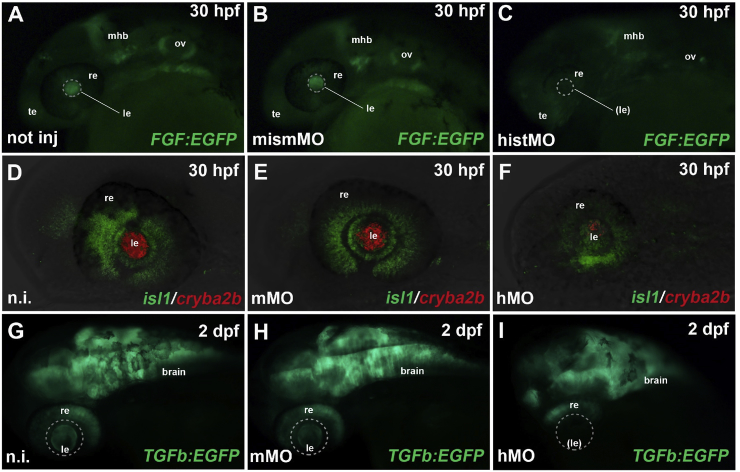
***Hist2h3ca1* knock-down affects zebrafish eye development.** a–c: After MO-mediated knockdown of zebrafish *hist2h3ca1*, FGF signaling (green EGFP reporter) was still preserved in telencephalic (te), otic vesicle (ov) and midbrain-hindbrain-boundary (mhb) regions, but lost in the lens (dashed circle) of morphant embryos (c), compared to not injected (a) and mismMO-injected controls (b), analyzed at 30 hpf (hours post-fertilization). re: retina. d–f: At 30 hpf, expression of *cryba2b* (*red*) was almost completely lost in the lens (le) of morphants (hMO) (f), compared to not injected (n.i.) (d) and mismatched (mMO) (e) controls, while *isl1* expression (green) was still present in the retina (re). g–i: TGFb (TGFβ) signaling (green EGFP reporter) was activated in the brain, retina (re) and lens (le) of not injected and control-injected embryos (g, h), while it was specifically absent in the lens region (dashed circle) of morphant embryos (i) at 2 dpf (day post-fertilization). All panels display lateral views of zebrafish cephalic regions, with anterior to the left. Displayed phenotypes are representative of n = 60 embryos per condition. The scale bars are 100 μm in A and G, 50 μm in D and apply to all images in the same row.

The zebrafish database (www.zfin.org/) indicates expression of the homologous zebrafish gene, *hist2h3ca1,* in the eye and in many proliferating tissues. Therefore, we used the zebrafish as a model organism to determine, if the role of *Hist2H3c1* in ocular development is conserved. Downregulation of the zebrafish *hist2h3ca1* gene by antisense morpholino oligomers (“morphant embryos”) led to developmental delay and to a specific ocular phenotype, similar that observed in the *Aey69* mouse. As shown in Fig. 12 (a, b, c), injection of anti-*hist2h3ca1* morpholinos in a transgenic line, reporting the activation of FGF signaling, a key pathway for lens induction and development in vertebrates (Garcia et al., 2011), led to a strong and specific decrease of reporter fluorescence in the prospective lens region at 1 day post fertilization (dpf). Analysis of retinal (*isl1*) and lens (*cryba2b*; βA2-crystallin) markers in the optic region of morphant embryos, at the same stage, reveals a dramatic decrease of *cryba2b* expression, while *islet-1* expression is relatively spared and particularly intense in the retinal ganglion layer (Fig. 12d, e, f), suggesting impaired differentiation specifically in the lens placode. Retinal differentiation was further assessed by knocking down *hist2h3ca1* in zebrafish transgenic lines labelling specific retinal cell types (Pax6b-, Ptf1a-, NeuroD- and Notch-signaling reporters), confirming the presence of a delayed and collapsed but still layered retina (outer and inner nuclear layer, retinal ganglion layer) (Supplementary Table S4 and shown for Ptf1a and NeuroD in Fig. S4). The perturbation of the lens development was verified also at 2 dpf, using a transgenic line for TGFβ signaling, a key pathway for lens formation and terminal differentiation (de Iongh et al., 2001). Indeed, the TGFβ signal appears correctly activated in the lens epithelium and in the lens fibers of control embryos. Conversely, the reporter fluorescence, while maintained in the retina, was essentially absent in the whole lens region in morphants (Fig. 12g, h, i). Collectively, these data suggested that lack of *hist2h3ca1* activity in zebrafish specifically affects lens development, while relatively sparing retinal formation and layering.

**Fig. 13:**
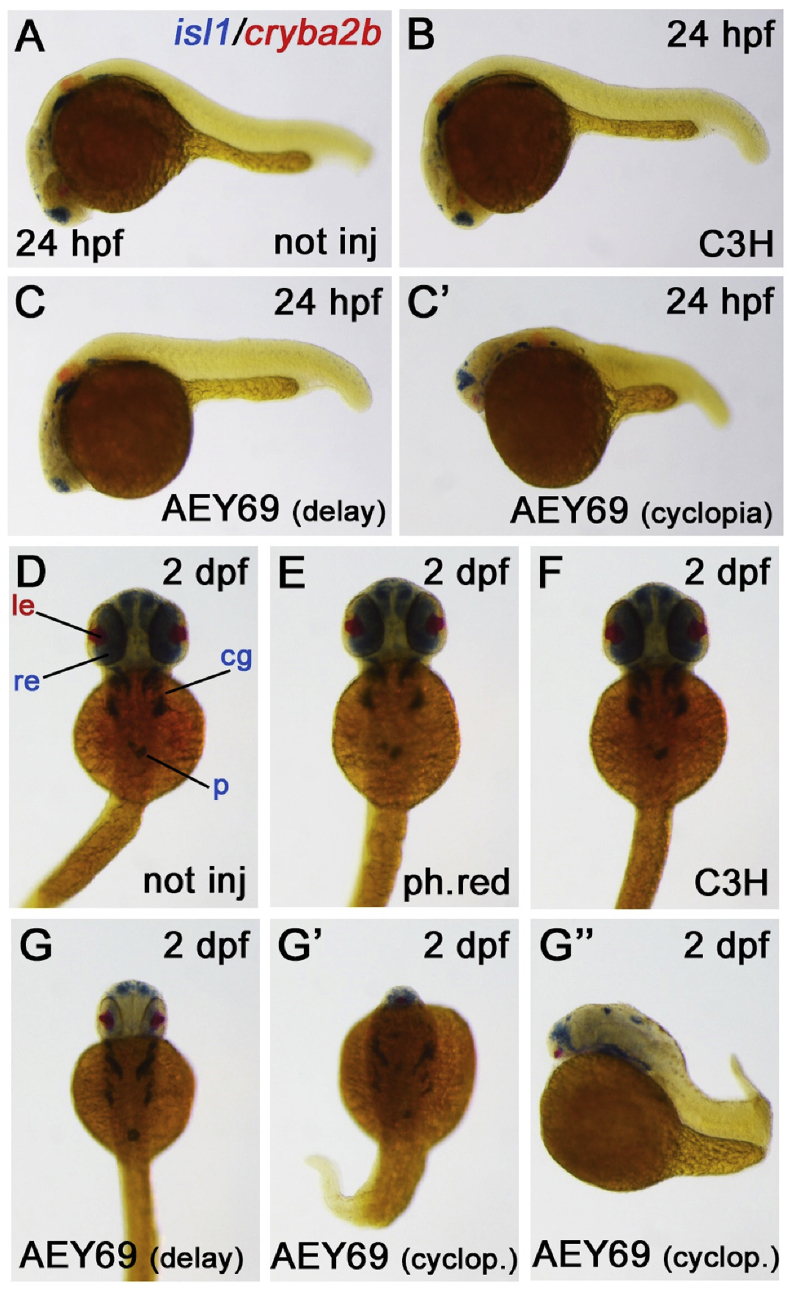
**Mutated *Hist2h3c1* over-expression perturbs zebrafish development and eye formation.** a-c’: Injection of mutated AEY69 *Hist2h3c1* mRNA into zebrafish embryos led to developmental delay (c), malformation and cyclopia (c’), while wild-type C3H mRNA was not eliciting any defect (b), compared to not injected controls (a), when analyzed at 24 hpf. d-g’‘: The analysis at 2 dpf confirmed normal and comparable phenotypes in not injected (d), control phenol-red-injected (ph.red) (e) and C3H-injected (f) embryos, while AEY69-injected embryos showed developmental delay (g), malformation and cyclopia (g’, g’‘). The *isl1* marker (blue) labels retina (re), cranial ganglia (cg) and pancreas (p), while *cryba2b* (red) identifies the lens (le) region. a-c’ and g’’ panels display lateral views with anterior to the left; d-g’ panels display dorsal views with anterior to the top. Displayed phenotypes are representative of n = 60 embryos per condition. Scale bar in A is 200 μm and applies to all images.

In addition, the injection of the *Aey69*-*Hist2h3c1*-mRNA into zebrafish embryos led to dramatic changes in eye morphology, including size reduction and, at higher dosages, failed separation of the eye field (cyclopia) while, at the same dose, the wild-type C3H mRNA did not elicit any abnormal phenotype (Fig. 13). Of note, the wild-type C3H mRNA rescued quite well the morpholino effects, in terms of viability and general morphology, while the mutated *AEY6*9 mRNA, at the same dose, did not compensate the morpholino activity, but instead exacerbates the morphant phenotype (Fig. S5 and Table S5).

Overall, these experiments suggested a conserved role for Hist2h3c1 proteins throughout the evolution, and that the c.358 A > C mutation has disrupting effects on vertebrate ocular development. Since the Ile120 position is conserved in many histone H3 subtypes and variants (Hake and Allis, 2006; Shi et al., 2011), we hypothesize a conserved role for this particular amino acid position.

## 4 Discussion

### 4.1 *Aey69* – a unique mouse model

We describe here a new mouse mutant, *Aey69*, with severe microphthalmia. The pathology of microphthalmia begins at embryonic stage of E11.5 similar to the *aphakia* mutant mice, when the mutant lens vesicle does not separate properly from the surface ectoderm (Fig. 2b). In *aphakia* mice, two major deletions in the *Pitx3* promoter are responsible for this defect (Semina et al., 2000; Rieger et al., 2001). However, in the early stages of *Aey69* development, the expression of PITX3 is maintained and therefore not responsible for the failed surface ectoderm separation (Fig. 10a). Moreover, *Pitx3* mutants show a loss of dopaminergic neurons in the *substantia nigra* with accompanying increased anxiety-related behavior and reduced locomotor activity (Rosemann et al., 2010). However, no such loss of dopaminergic neurons (Fig. S6) was observed in the *Aey69* mutants. Thus, the *Aey69* mutants represent a microphthalmic mouse model with unique underlying changes in embryonic eye development and diverse phenotypic defects in adult mice.

### 4.2 Early lens disappearance and retinal hyperproliferation leading to microphthalmia

The earliest hint for the ocular specification in the mouse happens at around E8.5 through the evagination of the diencephalon to form the lens placode. Subsequently, the lens vesicle is established at E11.5 following separation from the surface ectoderm (Smith et al., 2009). By contrast for the mutant lenses, it is clear that while proliferative cells can be detected until E13.5, there is a failure of lens fiber cell differentiation from E11.5 onwards. Thus for the lens, the most parsimonious explanation for the marker expression patterns and the phenotype documented is that there is a failure of primary fiber cell formation. The AP2α, PITX3, FOXE3 staining all indicate that from E11.5-E13.5 there are still epithelial cells that undergo proliferation (KI67). However, the crystallin expression and the cleaved caspase-3 stain indicate that early differentiating fibers are undergoing apoptosis. The progressive demise of the epithelial cells is more difficult to explain but may be associated with a failure of stem cell renewal, whereby all cells are pushed to enter G0 and then undergo apoptosis.

On the other hand, the immunohistochemical characterization of the retina through E11.5-E15.5 suggest a different story. In the wild type, retinal ganglion cells (RGC) are generated first, followed by cone photoreceptors and horizontal cells. After birth, bipolar cells and Müller glia are specified and complete differentiation (Zagozewski et al., 2014). In *Aey69*, these early retinal cell types, RGCs (BRN3; Fig. 10a) and cone photoreceptors (OTX2; Fig. 10b) were present from E11.5 onwards. There is an early appearance and over expression of these cell types in the mutant retina. This earlier expression of retinal cell types is accompanied by a strong retinal proliferation as seen by Ki67 staining at E15.5 (Fig. 11b). We speculate that this proliferation drives retinal growth, similar to a tumor, to occupy the vitreous and lens spaces (Fig. 11a). Thus, we see that the failed surface ectoderm separation has a pathological effect on both, the lens and the retina.

### 4.3 Mutant genes of *Aey69*

*Aey69* represents a unique mouse model with two point mutations in two diverse genes: a gap junction mediating intercellular communication (*Gja8*) and a histone gene providing structural and regulatory components for epigenetic regulation (*Hist2h3c1*). In the ocular lens, gap junction proteins (usually referred to as connexins) represent a key component of homeostatic mechanisms in maintenance of lens structure and transparency (Rubinos et al., 2014). The *Gja8* mutation in this mutant line (71 T- > C; Val24Ala) affects the first transmembrane domain. Dominant point mutation in *Gja8* have been reported in sites preceding this amino acid position and domain, namely G22R in *Lop10* mouse (Runge et al., 1992) and R23T (human) (Alapure et al., 2012). Both mutant forms are associated with a cataractous phenotype and smaller lenses (but not with no lens phenotype as in *Aey69*). In *Aey69*, the phenotype is much stronger and starts much earlier than the reported *Gja8* mutations. In addition, even in the similar phenotype, *aphakia*, GJA8 remains present in the mutant lens at E12.5, but it is absent in the *Aey69* mutants (Fig. 7). It might be speculated that the *Gja8* mutation in the *Aey69* mutant leads to a loss of the protein due to nonsense-mediated decay or mis-targeting of the protein from the endoplasmatic reticulum. However, since Gja8 is present in a few anterior cells at E11.5 (Fig. 7, arrows), this hypothesis does not seem to be very likely, and the missing Gja8 at E12.5 is explained rather by a secondary effect due to the disappearance of the lens. This led us to conclude that the primary role behind the *Aey69* phenotype is the *Hist2h3c1* mutation.

The *Hist2h3c1*-encoded canonical variant H3.2 is synthesized in a replication-dependent manner and has been found to occupy heterochromatic sites in mouse embryos throughout the preimplantation stage, i.e. from the one-cell stage through the blastocyst stage. This expression is a prerequisite to achieve the epigenetic reprogramming required for development (Akiyama et al., 2011). Apart from this observation, no specific role is known about *Hist2h3c1* and its encoded protein during development and in different tissues. There is high nucleotide conservation amongst the genes encoding for H3.2. Therefore, highly specific primer sequences were used to analyze the expression of the histone cluster genes in different tissues and across different embryonic stages. *Hist2h3c1* was found to be the most highly expressed histone H3.2 gene in the lens as compared to the other analyzed tissues of like retina, brain and liver (Fig. 4a). In addition, the gene was found to be down-regulated in the embryonic stages of the mutant (Fig. 4b). Therefore, it could be hypothesized that the *Hist2h3c1*-encoded H3.2 has an indispensable role in ocular development because of its increased expression in the adult wild-type lenses and dysregulated expression from E10.5-E12.5 in mutant embryos, when microphthalmia begins. While the mechanism is unclear, it is plausible that the Ile120Leu mutation in the H3.2 protein sequesters a critical lens regulatory protein and functions via dominant negative mechanism.

### 4.4 Evolutionary conservation of *Hist2h3c1* function

Our studies performed in the zebrafish system have corroborated the important role of *Hist2h3c1* during eye development. The over-expression experiments provide strong evidence that the identified *Hist2h3c1* mutation acts in a dominant fashion, as the mutant but not the wild-type mouse mRNA strongly perturbs the ocular development when over-expressed in zebrafish embryos. Interestingly, the knock-down of the endogenous *hist2h3ca1* gene in zebrafish also impaired eye development, eliciting lens-specific disrupting effects. According to the ZFIN database, the zebrafish *hist2h3ca1* gene has a strong ocular expression, but it is also expressed in other proliferative tissues. These observations on zebrafish *hist2h3ca1* expression are strongly in line with the *Aey69* microphthalmic phenotype and with the additional impairments in other organs and systems (fat content, body temperature, hematological and immunological parameters; Table 1). In summary, while a possible role for *Gja8* in modulating the *Aey69* phenotype cannot be totally excluded, the temporal and spatial pattern of *Hist2h3c1* makes this locus a more likely causative gene for the observed phenotype.

### 4.5 Conclusion, speculation and future outlook

The mutation in the *Hist2h3c1* gene (c.358 A > C, Ile120Leu) affects the loop region of H3.2 (Tropberger and Schneider, 2010). Since modelling of the wild-type and mutant proteins suggested structural divergence, it might be speculated that this mutation site affects the diverse posttranscriptional modifications of the protein. Though the regulatory role of posttranscriptional modifications of the histone H3 family, particularly histone H3 K9 acetylation, has been well characterized in lens specification (Yang et al., 2006), however, to the best of our knowledge, no study on the nature of specific histone H3 subtypes carrying these modifications has been published. The seemingly slight differences in sequence between H3 isoforms could mean that the histone isoforms are interchangeable in their function. However, epigenetic experiments established that the structurally conserved mammalian histone H3 variants (H3.1, H3.2, and H3.3) exhibit distinct posttranscriptional modifications, which influence epigenetic states during cellular differentiation and development (Hake and Allis, 2006). Subsequently, theoretical concepts of histone gene expression in regulating differentiation have been developed (Maehara et al., 2015), and our initial documentation of the mutated *Hist2h3c1* gene in the microphthalmic *Aey6*9 might open further avenues for more detailed studies.

*Hist2h3c1* represents one of the evolutionary conserved mammalian histone genes. Owing to the nucleotide conservation amongst the gene isoforms and the structural similarity between the various histone H3 subtypes, biochemical elucidation of the exact role of the *Hist2h3c1* gene and its protein during embryonic development is hard to analyze. Nevertheless, the pathophysiological characterization including the disappearing lens vesicle and the hyperproliferation of the retina in the *Aey69* mouse mutant added already valuable insights into the function of this particular histone H3.2. The future characterization of specific properties of the core histone H3.2 through ChIP Seq, NoMEseq or H3.2-specific interactomics in this unique mutant line will deepen our understanding of the functions of histone H3.2 during eye development.

## Conflicts of interest

None declared.

## Funding

SV is supported by the German Academic Exchange Service (DDAD; Grant ID 57129429); NT is supported by the AFM Telethon project POLYGON (18572), by the Italian Ministry of Health grant RF-2010-2309484, and by the University of Padova projects OPTOZEN (CPDA128151) and TIGRE (CPDA148582). This work was supported from the Helmholtz Portfolio Theme ‘Metabolic Dysfunction and Common Disease’ (JB) and the Helmholtz Alliance ‘Aging and Metabolic Programming, AMPro’ (JB) and by the German Federal Ministry of Education and Research (Infrafrontier grant 01KX1012) (MHA).

## Glossary

Aey69: abnormality of the eye #69
AP2α: Activating enhancer-binding protein 2α
ARVO: Association for Research in Vision and Ophthalmology
ASAT: Aspartate aminotransferase
BRN3: Brain-Specific Homeobox 3
CALBINDIN: Vitamin D-Dependent Calcium-Binding Protein, Avian-Type
CEF3: Translation elongation factor EF-3
*Cgn*: Cingulin
*c-Maf*: Avian Musculoaponeurotic Fibrosarcoma (MAF) Protooncogene
*Cryaa*: Crystallin, αA
*Cryba2b*: Crystallin, βA2
*Crygd*: Crystallin, γD
DAPI: 4′,6-Diamidino-2-Phenylindole, Dihydrochloride
DHEA: Dehydroepiandrosteron
dpf: Days post fertilization
DUSP6: Dual Specificity Phosphatase 6
EGFP: Enhanced Green Fluorescent Protein
ENU: Ethyl nitroso urea
FOXE3: forkhead box E3
GCL: ganglion cell layer
GFAP: Glial Fibrillary Acidic Protein
GMC: German Mouse Clinic
HDL: High-density lipoprotein
Ig: Immunoglobulin
Inbl: Inner neuroblastic layer
IpGTT: Intraperitoneal Glucose Tolerance Test
ISL1: Insulin gene enhancer protein
*I-TASSER*: Iterative Threading ASSEmbly Refinement
KAT3: Histone Lysine Acetyltransferases
LDH: Lactate-dehydrogenase
LE: Lens Epithelium
*Lhx2*: LIM/homeobox protein 2
Me: Methylation
NMR: Nuclear Magnetic Resonance
NR: Neural Retina
ONBL: Outer Neuroblastic Layer
*OTX2*: Orthodenticle Homeobox 2
ONBL: Paired box gene 2
PBS: Phosphate Buffered Saline
PFA: Paraformaldehyde
Phospho: Phsophorylation
Pitx3: Pituitary homeobox 3
PKCA: Protein Kinase C-Alpha
*Pogz*: Pogo transposable element with ZNF domain
PTU: Propylthiouracil
Pymol: Python-enhanced molecular graphics
REST: Relative expression software tool
RGC: Retinal Ganglion Cells
RMSD: Root Mean Square Deviation
RPC: Retinal Progenitor Cells
RPE: Retinal Pigmented Epithelium
Rx: Retinal homeobox protein
SHIRPA: SmithKline Beecham, Harwell, Imperial College, Royal London Hospital, phenotype assessment
SOX2: SRY (sex determining region Y)-box 2
TH: Tyrosine hydroxylase
TZ: Transition Zone
WISH: Whole-mount *in-situ* hybridization

## Appendix A

^§^German Mouse Clinic, Helmholtz Zentrum München, German Research Center for Environmental Health GmbH, Neuherberg, Germany

Thure Adler^1,2,∗^

Irina Treise^1,2^

Dirk H. Busch^2^

Antonio Aguilar-Pimentel^1,3^

Markus Ollert^3,11^

Alexander Götz^1,4,+^

Oana V Amarie^1,4,#^

Tobias Stoeger^1,4^

Holger Schulz^1,4,∗∗^

Lore Becker^1,5^

Thomas Klopstock^5,16,20,21^

Anja Schrewe^1^

Nadine Spielmann^1^

Raffi Bekeredjian^6^

Lillian Garrett^1,8^

Sabine M. Hölter^1,8^

Annemarie Zimprich^1,8^

Wolfgang Wurst^8,14,15,16,17^

Philipp Mayer-Kuckuk^1^

Wolfgang Hans^1^

Jan Rozman^1,19^

Martin Klingenspor^9,10^

Frauke Neff^1,7^

Patricia da Silva-Buttkus^1^

Julia Calzada-Wack^1^

Ildikó Rácz^1,12,^“

Andreas Zimmer^12^

Birgit Rathkolb^1,13,19^

Eckhard Wolf^13^

Cornelia Prehn^1^

Jerzy Adamski^1,18^

Manuela Östereicher^1^

Gregor Miller^1^

Ralph Steinkamp^1^

Christoph Lengger^1^

Holger Maier^1^

Claudia Stoeger^1^

Stefanie Leuchtenberger^1^

Valérie Gailus-Durner^1^

Helmut Fuchs^1^

Martin Hrabě de Angelis^1,18,19^

1. German Mouse Clinic, Institute of Experimental Genetics, Helmholtz Zentrum München, German Research Center for Environmental Health GmbH, Ingolstädter Landstrasse 1, 85764 Neuherberg, Germany

2. Institute for Medical Microbiology, Immunology and Hygiene, Technical University of Munich, Trogerstrasse 30, 81675 Munich, Germany

3. Department of Dermatology and Allergy, Biederstein, Klinikum rechts der Isar, Technische Universität München (TUM), Biedersteiner Str. 29, 80802 Munich,

4. Comprehensive Pneumology Center, Institute of Lung Biology and Disease, Helmholtz Zentrum München, German Research Center for Environmental Health (GmbH), Ingolstädter Landstraβe 1, 85764 Neuherberg, Germany and Member of the German Center for Lung Research

5. Department of Neurology, Friedrich-Baur-Institut, Ludwig-Maximilians-Universität München, Ziemssenstrasse 1a, 80336 Munich, Germany

6. Department of Cardiology, University of Heidelberg, Im Neuenheimer Feld 410, 69120 Heidelberg, Germany

7. Institute of Pathology, Helmholtz Zentrum München, German Research Center for Environmental Health GmbH, Ingolstädter Landstrasse 1, 85764 Neuherberg, Germany

8. Institute of Developmental Genetics, Helmholtz Zentrum München, German Research Center for Environmental Health GmbH, Ingolstädter Landstrasse 1, 85764 Neuherberg, Germany

9. Chair for Molecular Nutritional Medicine, Technische Universität München, Else Kröner-Fresenius Center for Nutritional Medicine, 85350 Freising, Germany

10. ZIEL – Center for Nutrition and Food Sciences, Technische Universität München, 85350 Freising, Germany

11. Department of Infection and Immunity, Luxembourg Institute of Health, Esch-sur-Alzette, Luxembourg, and Department of Dermatology and Allergy Center, Odense Research Center for Anaphylaxis, University of Southern Denmark, Odense, Denmark

12. Institute of Molecular Psychiatry, University of Bonn, Sigmund-Freud-Strasse 25, 53127 Bonn, Germany

13. Ludwig-Maximilians-Universität München, Gene Center, Institute of Molecular Animal Breeding and Biotechnology, Feodor-Lynen Strasse 25, 81377 Munich, Germany

14. Chair of Developmental Genetics, Center of Life and Food Sciences Weihenstephan, Technische Universität München, Ingolstädter Landstrasse 1, 85764 Neuherberg, Germany

15. Max Planck Institute of Psychiatry, Kraepelinstr. 2-10, 80804 Munich, Germany

16. Deutsches Institut für Neurodegenerative Erkrankungen (DZNE) Site Munich, Schillerstrasse 44, 80336 Munich, Germany

17. Munich Cluster for Systems Neurology (SyNergy), Adolf-Butenandt-Institut, Ludwig-Maximilians-Universität München, Schillerstrasse 44, 80336 Munich, Germany

18. Chair of Experimental Genetics, Center of Life and Food Sciences Weihenstephan, Technische Universität München, 85354 Freising-Weihenstephan, Germany

19. Member of German Center for Diabetes Research (DZD), Ingolstädter Landstraβe 1, 85764 Neuherberg, Germany

20. German Network for Mitochondrial Disorders (mitoNET)

21. German Center for Vertigo and Balance Disorders, Munich, Germany

^∗^ present address: Comprehensive Pneumology Center (CPC), Helmholtz Zentrum München, German Research Center for Environmental Health GmbH, Ingolstädter Landstrasse 1, 85764 Neuherberg, Germany.

^+^ present address: Institut für Neuropathologie, Universitätsmedizin Göttingen (UMG), Robert-Koch-Str. 40, 37099 Göttingen, Germany.

^#^ present address: Institute of Developmental Genetics, Helmholtz Zentrum München, German Research Center for Environmental Health GmbH, Ingolstädter Landstrasse 1, 85764 Neuherberg, Germany.

^∗∗^ present address: Institute of Epidemiology, Helmholtz Zentrum München, German Research Center for Environmental Health GmbH, Ingolstädter Landstrasse 1, 85764 Neuherberg, Germany.

“ present address: Clinic of Neurodegenerative Diseases and Gerontopsychiatry, University of Bonn Medical Center, Bonn, Germany.
