# Supplementary material for: Mutation in the mouse histone gene Hist2h3c1 leads to degeneration of the lens vesicle and severe microphthalmia
Source: Exp Eye Res. 2019 Nov;188:107632. doi: 10.1016/j.exer.2019.03.024 (PMC6876282; doi:10.1016/j.exer.2019.03.024)
Supplement: Multimedia component 1 [file mmc1.docx]

Supplement

**Supplementary Table S1**

**PCR Primers**

| **Name** | **Sequence 5’ 🡪 3’*** | **Annealing temperature [^o^C]** | **Product**  **size [bp]** |
| --- | --- | --- | --- |
| *Aey69*-L1 | TACCAGAAGTCGACCGAGCTGC | 67 | 463 |
| *Aey69*-R1 | TCACTGCGCTCGCAATTGC |  |  |
| *Hist2h3c1*-L1 | CTTGCCTTAGTTAACACCCTCA | 60 | 100 |
| *Hist2h3c1*-R1 | TTCAAGATTCTCCTTCTAGATTGTTAC |  |  |
| *H3*-all-L1 | TAAATCCACTGTGATATCGACTTCAAGACCGACCTGCGCTT | 60 | 100 |
| *H3*-all-R1 | ATTATGCTGAGTGATATCTCGTGTGTGGCTCTGAAAAGAGCCT |  |  |
| *Hist1h3b*-L1 | GCAGGACTTCAAGACCGACC | 60 | 195 |
| *Hist1h3b*-R1 | GCACTTTGTTTCGTTTAAGCCC |  |  |
| *Hist1h3c*-L1 | ACTTTTGTGTATCTCCTCCCAAG | 60 | 150 |
| *Hist1h3c*-R1 | GTAGCGGTGAGGCTTCTTCA |  |  |
| *Hist1h3d* -L1 | ACCGTTCTCATTCCCTGAGAC | 60 | 165 |
| *Hist1h3d*-R1 | GTAGCGGTGAGGCTTCTTCA |  |  |
| *Hist1h3f*-L1 | CCGCTGTGGTATCGCCAA | 60 | 109 |
| *Hist1h3f*-R1 | ACTATTGCTTCCTCAGCCAC |  |  |
| *Hist2h3c*2-R1 | CACATTATCCCGCTCCATAGCTCTAG | 60 | 118 |
| *Hist2h3c2*-L1 | CAAGCTAGGAGTCTGAATAAGACCGC |  |  |
| *Hist2h3b*-L1 | GTATGGCCACTTGGCGTCTG | 60 | 166 |
| *Hist2h3b*-R1 | GTAGCGGTGCGGCTTCTTC |  |  |
| *Rplp0*-L1 | ACTGGTCTAGGACCCGAGAAG | 60 | 77 |
| *Rplp0*-R1 | TCAATGGTGCCTCTGGAGATT |  |  |

**Morpholino Oligomers**

| histMO | CGGTCTGCTTGGTTCTTGCCATAGC |
| --- | --- |
| mismMO | CGcTCTcCTTcGTTgTTcCCATAGC |

Lower-case bases indicate the five mutated bases in the non-functional mismMO, to be compared with the correct sequence of the working histMO morpholino.

**Supplementary Table S2**

**Antibodies**

| **Antibody** | **Company (catalog number)** | **Dilution** |
| --- | --- | --- |
| AP-2α | Santa Cruz (sc-12726) | 1:100 |
| BRN3 | Santa Cruz (sc-6026) | 1:100 |
| Calbindin | Swant (CB38a) | 1:500 |
| Cleaved Caspase-3 | Cell signalling (9661S) | 1:100 |
| Crystallin, αA | Dr. Ales Cvekl, NY/USA  (Xie et al., 2016) | 1:500 |
| Crystallin, γD | Santa Cruz (sc-22746) | 1:500 |
| Cx50/GJA8 | Thermo Scientific (PA511644) | 1:500 |
| FOXE3 | Santa Cruz (sc-134536) | 1:100 |
| Glial Fibrillary Acidic Protein | Sigma (G9269) | 1:100 |
| Ki67 | Abcam (ab15580) | 1:100 |
| OTX2 | Abcam (ab114138) | 1:100 |
| PITX3 | Santa Cruz (sc-19307) | 1:100 |
| Protein Kinase Cα | Abcam (ab11723) | 1:200 |
| TH | Pel Freez (P40101-150) | 1:1000 |
| Alexa Fluor® 488 Rabbit | Invitrogen (A-21206) | 1:250 |
| CY3 Goat | Jackson immuno (705-165-147) | 1:250 |
| CY3 Mouse | Jackson immuno (715-165-150) | 1:250 |

Xie,Q., McGreal,R., Harris,R., Gao,C.Y., Liu,W., Reneker,L.W., Musil,L.S., Cvekl,A. (2016) Regulation of c-Maf and αA-crystallin in ocular lens by fibroblast growth

factor signaling. *J. Biol. Chem.* **291**, 3947-3958.

**Supplementary Table S3**

**Relevant Significant Parameters of GMC investigation**

Depicted are the number of animals, the mean and the standard deviation of the observations and the p-value of the statistical tests. The Fishers exact test deals with categorical data and the number of attributes and the p-values are listed.

| \| Parameters analyzed by linear model with sex, genotype and body weight as predictors \| \| \| \| \| \| \|  \| \|  \| \|  \| \|  \| \|  \| \|  \| \| \| --- \| --- \| --- \| --- \| --- \| --- \| --- \| --- \| --- \| --- \| --- \| --- \| --- \| --- \| --- \| --- \| --- \| --- \| --- \| \|  \| \| \| \| \| \| \|  \|  \| \|  \| \|  \| \|  \| \|  \| \| \| test \| parameter \| f.muts \| f.cons \| m.muts \| m.cons \| sex \| genotype \| weight \| \| sex.genotype \| \| sex.weight \| \| genotype. weight \| \| sex.genotype_weight \| \| \| Calorimetry \| body_temp (°C) \| 7 (38 ± 0.4 ) \| 7 (37 ± 0.4 ) \| 7 (37 ± 0.75 ) \| 7 (37 ± 0.36 ) \| 0.442 \| 0.388 \| 0.041 \| \| 0.029 \| \| 0.583 \| \| 0.325 \| \| 0.043 \| \|  \| Parameters analyzed by linear model with sex and genotype as predictors \| \| \| \| \| \|  \|  \|  \| \| --- \| --- \| --- \| --- \| --- \| --- \| --- \| --- \| --- \| \| test \| parameter \| f.muts \| f.cons \| m.muts \| m.cons \| sex \| genotype \| sex.genotype \| \| Clinical_ Chemistry \| Albumin_ mean \| 10 (28 ± 0.96 ) \| 10 (29 ± 0.63 ) \| 10 (28 ± 0.76 ) \| 10 (27 ± 0.79 ) \| <0.001 \| 0.081 \| 0.008 \| \| Clinical_ Chemistry \| Cl_mean \| 10 (111 ± 0.68 ) \| 10 (109 ± 0.5 ) \| 10 (108 ± 1.1 ) \| 10 (108 ± 0.95 ) \| <0.001 \| 0.001 \| 0.001 \| \| Clinical_ Chemistry \| GOT_mean \| 10 (53 ± 19 ) \| 10 (44 ± 3.2 ) \| 10 (71 ± 21 ) \| 10 (47 ± 6.1 ) \| 0.041 \| 0.001 \| 0.116 \| \| Clinical_ Chemistry \| LDH_mean \| 10 (200 ± 57 ) \| 10 (158 ± 20 ) \| 10 (368 ± 114 ) \| 10 (253 ± 27 ) \| <0.001 \| 0.001 \| 0.09 \| \| SHIRPA \| locomotor_ activity \| 10 (15 ± 7.4 ) \| 10 (12 ± 7.9 ) \| 10 (21 ± 11 ) \| 10 (12 ± 8.6 ) \| 0.377 \| 0.049 \| 0.232 \| \| OPEN_FIELD \| DisTTot \| 10 (15033 ± 2321 ) \| 10 (9978 ± 2689 ) \| 10 (14056 ± 2451 ) \| 10 (8889 ± 1725 ) \| 0.169 \| <0.001 \| 0.94 \| \| OPEN_FIELD \| NRTot \| 10 (78 ± 24 ) \| 10 (52 ± 37 ) \| 10 (71 ± 34 ) \| 10 (27 ± 20 ) \| 0.112 \| 0.001 \| 0.343 \| \| OPEN_FIELD \| PcTiCenTot \| 10 (11 ± 6.3 ) \| 10 (2.4 ± 2.1 ) \| 10 (10 ± 10 ) \| 10 (3.4 ± 3.5 ) \| 0.849 \| 0.001 \| 0.776 \|  \| Parameters analyzed by Wilcoxon test \| \| \| \| \| \| \|  \| \|  \| \|  \| \|  \| \|  \| \|  \| \| \| --- \| --- \| --- \| --- \| --- \| --- \| --- \| --- \| --- \| --- \| --- \| --- \| --- \| --- \| --- \| --- \| --- \| --- \| --- \| \| tests \| \| parameter \| \| Number.f.muts \| \| \| Number.f.cons \| \| Number.m.muts \| \| Number.m.cons \| \| p.female \| \| p.male \| \| p.overall \| \| \| IMMUNOGLOBULIN \| \| IgA \| \| 8 (340 ± 132 ) \| \| \| 9 (612 ± 369 ) \| \| 10 (236 ± 83 ) \| \| 10 (239 ± 162 ) \| \| 0.036 \| \| 0.315 \| \| 0.62 \| \| \| FACS_BLOOD \| \| CD8+ T cells (% of all living leukocytes) \| \| 9 (12 ± 1.9 ) \| \| \| 8 (10 ± 1.8 ) \| \| 10 (12 ± 1.7 ) \| \| 10 (11 ± 1.7 ) \| \| 0.044 \| \| 0.447 \| \| 0.074 \| \| \| FACS_BLOOD \| \| Granulocytes (% of all living leukocytes) \| \| 9 (20 ± 4.1 ) \| \| \| 8 (24 ± 3.9 ) \| \| 10 (18 ± 5.8 ) \| \| 10 (24 ± 7.1 ) \| \| 0.059 \| \| 0.105 \| \| 0.008 \| \| \| FACS_BLOOD \| \| Monocytes (% of all living leukocytes) \| \| 9 (1.8 ± 0.54 ) \| \| \| 8 (1.2 ± 0.24 ) \| \| 10 (2.2 ± 0.55 ) \| \| 10 (2 ± 0.48 ) \| \| 0.007 \| \| 0.353 \| \| 0.074 \| \| \| FACS_BLOOD \| \| CD11+ NK cells (% of NK cells) \| \| 9 (64 ± 6.2 ) \| \| \| 8 (58 ± 3.5 ) \| \| 10 (63 ± 13 ) \| \| 10 (59 ± 6 ) \| \| 0.034 \| \| 0.436 \| \| 0.038 \| \| \| FASTED_BLOOD \| \| Glycerol_fasting \| \| 10 (0.35 ± 0.074 ) \| \| \| 10 (0.38 ± 0.052 ) \| \| 10 (0.25 ± 0.042 ) \| \| 10 (0.31 ± 0.045 ) \| \| 0.353 \| \| 0.019 \| \| 0.034 \| \| \| FASTED_BLOOD \| \| HDL_fasting \| \| 10 (2.3 ± 0.2 ) \| \| \| 10 (2.7 ± 0.23 ) \| \| 10 (3.5 ± 0.18 ) \| \| 10 (3.4 ± 0.21 ) \| \| 0.002 \| \| 0.238 \| \| 0.525 \| \| \| FASTED_BLOOD \| \| Non_HDL_chol_fasting \| \| 10 (0.52 ± 0.11 ) \| \| \| 10 (0.64 ± 0.11 ) \| \| 10 (0.76 ± 0.084 ) \| \| 10 (0.75 ± 0.071 ) \| \| 0.041 \| \| 1 \| \| 0.227 \| \| \| FASTED_BLOOD \| \| TG_fasting \| \| 10 (1.3 ± 0.69 ) \| \| \| 10 (2.3 ± 0.32 ) \| \| 10 (1.7 ± 0.49 ) \| \| 10 (1.9 ± 0.45 ) \| \| 0.002 \| \| 0.399 \| \| 0.001 \| \| \| FASTED_BLOOD \| \| Total_chol_fasting \| \| 10 (2.9 ± 0.29 ) \| \| \| 10 (3.3 ± 0.24 ) \| \| 10 (4.3 ± 0.22 ) \| \| 10 (4.2 ± 0.24 ) \| \| 0.002 \| \| 0.229 \| \| 0.541 \| \| \| IPGTT_201311 \| \| Glucose_conc_0 \| \| 10 (4.5 ± 0.58 ) \| \| \| 10 (3.9 ± 0.43 ) \| \| 8 (5.8 ± 0.42 ) \| \| 9 (5.1 ± 0.68 ) \| \| 0.012 \| \| 0.069 \| \| 0.046 \| \| \| STEROID \| \| DHEA \| \| 10 (46 ± 20 ) \| \| \| 9 (80 ± 56 ) \| \| 10 (215 ± 88 ) \| \| 10 (125 ± 63 ) \| \| 0.133 \| \| 0.023 \| \| 0.667 \| \| \| DEXA \| \| bw_pDEXA \| \| 10 (27 ± 3.1 ) \| \| \| 10 (30 ± 3.2 ) \| \| 10 (34 ± 2.1 ) \| \| 10 (33 ± 4.1 ) \| \| <0.001 \| \| 0.201 \| \| 0.083 \| \| \| DEXA \| \| FAT_MASS \| \| 10 (3.7 ± 2 ) \| \| \| 10 (7.9 ± 1.6 ) \| \| 10 (9.7 ± 4 ) \| \| 10 (9.6 ± 5.5 ) \| \| 0.002 \| \| 0.087 \| \| 0.075 \| \| \| Fisher's Exact test \| \| \|  \| \|  \|  \| \|  \| \|  \| \|  \| \|  \| \|  \| \| \| test/ parameter \| Male \| \| \| \| Female \| \| \| Both \| \| \| \| \| \| \| \| \| \| \| SHIRPA \| Control \| \| Mutant \| \| Control \| Mutant \| \| Control \| \| Mutant \| \| Control \| \| Mutant \| \| p value \| \| \|  \| n=10 \| \| n=10 \| \| n=10 \| n=10 \| \| n=20 \| \| n=20 \| \| 50% \| \| 50% \| \|  \| \| \| *Palpebral Closure* \|  \| \| \| \| \| \| \| \| \| \| \| \| \| \| \| <0.001 \| \| \| Eyes open \| 10 \| \| 0 \| \| 10 \| 2 \| \| 20 \| \| 2 \| \| 100% \| \| 10% \| \|  \| \| \| Eyes closed \| 0 \| \| 10 \| \| 0 \| 8 \| \| 0 \| \| 18 \| \| 0% \| \| 90% \| \|  \| \| \| *Tail Elevation* \|  \| \| \| \| \| \| \| \| \| \| \| \| \| \| \| <0.001 \| \| \| Dragging \| 9 \| \| 2 \| \| 8 \| 3 \| \| 17 \| \| 5 \| \| 85% \| \| 25% \| \|  \| \| \| Horizontally extension \| 1 \| \| 8 \| \| 2 \| 7 \| \| 3 \| \| 15 \| \| 15% \| \| 75% \| \|  \| \| \| Elevated/Straub tail \| 0 \| \| 0 \| \| 0 \| 0 \| \| 0 \| \| 0 \| \| 0% \| \| 0% \| \|  \| \|  \| **Abbreviations:**   \| Bw \| Bodyweight \| \| --- \| --- \| \| Cl \| Chloride \| \| DHEA \| Dehydroepiandrosteron \| \| DisT \| Distance traveled \| \| GOT \| Glutamate oxaloacetate transaminase \| \| HP \| Heat production \| \| HDL \| High-density lipoprotein \| \| Ig \| Immunoglobulin \| \| IpGTT \| Intraperitoneal Glucose Tolerance Test \| \| LDH \| Lactate-dehydrogenase \| \| MV \| Minute ventilation \| \| NEFA \| Non-esterified fatty acid \| \| NR \| Number of rears \| \| PcTiCen \| Percentage time in center \| \| pDEXA \| peripheral dual-energy x-ray absorptiometry \| \| PEF \| Peak expiratory flow rate \| \| RER \| Respiratory exchange ratio \| \| SHIRPA \| SmithKline Beecham, Harwell, Imperial College, Royal London Hospital, phenotype assessment \| \| TG \| Triglyceride \| \| TV \| Tidal volume \| \| VO2 \| Oxygen consumption \| \| \|  \|  \| \| \| --- \| --- \| --- \| --- \| --- \| --- \| --- \| --- \| --- \| --- \| --- \| --- \| --- \| --- \| --- \| --- \| --- \| --- \| --- \| --- \| --- \| --- \| --- \| --- \| --- \| --- \| --- \| --- \| --- \| --- \| --- \| --- \| --- \| --- \| --- \| --- \| --- \| --- \| --- \| --- \| --- \| --- \| --- \| --- \| --- \| --- \| --- \| \|  \| \| \|  \| \|  \| |  |  |  |  |  |  |
| --- | --- | --- | --- | --- | --- | --- | --- | --- | --- | --- | --- | --- | --- | --- | --- | --- | --- | --- | --- | --- | --- | --- | --- | --- | --- | --- | --- | --- | --- | --- | --- | --- | --- | --- | --- | --- | --- | --- | --- | --- | --- | --- | --- | --- | --- | --- | --- | --- | --- | --- | --- | --- | --- | --- | --- | --- | --- | --- | --- | --- | --- | --- | --- | --- | --- | --- | --- | --- | --- | --- | --- | --- | --- | --- | --- | --- | --- | --- | --- | --- | --- | --- | --- | --- | --- | --- | --- | --- | --- | --- | --- | --- | --- | --- | --- | --- | --- | --- | --- | --- | --- | --- | --- | --- | --- | --- | --- | --- | --- | --- | --- | --- | --- | --- | --- | --- | --- | --- | --- | --- | --- | --- | --- | --- | --- | --- | --- | --- | --- | --- | --- | --- | --- | --- | --- | --- | --- | --- | --- | --- | --- | --- | --- | --- | --- | --- | --- | --- | --- | --- | --- | --- | --- | --- | --- | --- | --- | --- | --- | --- | --- | --- | --- | --- | --- | --- | --- | --- | --- | --- | --- | --- | --- | --- | --- | --- | --- | --- | --- | --- | --- | --- | --- | --- | --- | --- | --- | --- | --- | --- | --- | --- | --- | --- | --- | --- | --- | --- | --- | --- | --- | --- | --- | --- | --- | --- | --- | --- | --- | --- | --- | --- | --- | --- | --- | --- | --- | --- | --- | --- | --- | --- | --- | --- | --- | --- | --- | --- | --- | --- | --- | --- | --- | --- | --- | --- | --- | --- | --- | --- | --- | --- | --- | --- | --- | --- | --- | --- | --- | --- | --- | --- | --- | --- | --- | --- | --- | --- | --- | --- | --- | --- | --- | --- | --- | --- | --- | --- | --- | --- | --- | --- | --- | --- | --- | --- | --- | --- | --- | --- | --- | --- | --- | --- | --- | --- | --- | --- | --- | --- | --- | --- | --- | --- | --- | --- | --- | --- | --- | --- | --- | --- | --- | --- | --- | --- | --- | --- | --- | --- | --- | --- | --- | --- | --- | --- | --- | --- | --- | --- | --- | --- | --- | --- | --- | --- | --- | --- | --- | --- | --- | --- | --- | --- | --- | --- | --- | --- | --- | --- | --- | --- | --- | --- | --- | --- | --- | --- | --- | --- | --- | --- | --- | --- | --- | --- | --- | --- | --- | --- | --- | --- | --- | --- | --- | --- | --- | --- | --- | --- | --- | --- | --- | --- | --- | --- | --- | --- | --- | --- | --- | --- | --- | --- | --- | --- | --- | --- | --- | --- | --- | --- | --- | --- | --- | --- | --- | --- | --- | --- | --- | --- | --- | --- | --- | --- | --- | --- | --- | --- | --- | --- | --- | --- | --- | --- | --- | --- | --- | --- | --- | --- | --- | --- | --- | --- | --- | --- | --- | --- | --- | --- | --- | --- | --- | --- | --- | --- | --- | --- | --- | --- | --- | --- | --- | --- | --- | --- | --- | --- | --- | --- | --- | --- | --- | --- | --- | --- | --- | --- | --- | --- | --- | --- | --- | --- | --- | --- | --- | --- | --- | --- | --- | --- | --- | --- | --- | --- | --- | --- | --- | --- | --- | --- | --- | --- | --- | --- | --- | --- | --- | --- | --- | --- | --- | --- | --- | --- | --- | --- | --- | --- | --- | --- | --- | --- | --- | --- | --- | --- | --- | --- | --- | --- | --- | --- | --- | --- | --- | --- | --- | --- | --- | --- | --- | --- | --- | --- | --- | --- | --- | --- | --- | --- | --- | --- | --- | --- | --- | --- | --- | --- | --- | --- | --- | --- | --- | --- | --- | --- | --- | --- | --- | --- | --- | --- | --- | --- | --- | --- | --- | --- | --- | --- | --- | --- | --- | --- | --- | --- | --- | --- | --- | --- | --- | --- | --- | --- | --- | --- | --- | --- | --- | --- | --- | --- | --- | --- | --- | --- | --- | --- | --- | --- | --- | --- | --- | --- | --- | --- | --- | --- | --- | --- | --- | --- | --- | --- | --- | --- | --- | --- | --- | --- | --- | --- | --- | --- | --- | --- | --- | --- | --- | --- | --- | --- | --- | --- | --- | --- | --- | --- | --- | --- | --- | --- | --- | --- | --- | --- | --- | --- | --- | --- | --- | --- | --- | --- | --- | --- | --- | --- | --- | --- | --- | --- | --- | --- | --- | --- | --- | --- | --- | --- | --- | --- | --- | --- | --- | --- | --- | --- | --- | --- | --- | --- | --- | --- | --- | --- | --- | --- | --- | --- | --- | --- | --- | --- | --- | --- | --- | --- | --- | --- | --- | --- | --- | --- | --- | --- | --- | --- | --- | --- | --- | --- | --- | --- | --- | --- | --- | --- | --- | --- | --- | --- | --- | --- | --- | --- | --- | --- | --- | --- |

**Supplementary Table S4**

**Summary of *hist2h3ca1* knock-down experiments in zebrafish transgenic lines**

The table includes raw data on mortality and phenotype after *hist2h3ca1*-specific antisense morpholino (MO) injection in zebrafish transgenic lines.

mMO = control mismatched MO; hMO = anti-*hist2h3ca1* MO; n.e.=not evaluable.

| **Transgenic line** | **Condition** | **# embryos** | **Mortality**  **at 48 hpf** | **Morphology of survivors** | **Retinal organization** |
| --- | --- | --- | --- | --- | --- |
| *pax6b:GFP* | mMO 100 μM | 50 | 15% | 90% normal  10% apoptotic eyes and brain, short trunk | 90% normal  10% n.e. |
|  | hMO 100 μM | 50 | 100% | Lethality during gastrulation | n.e. |
| *ptf1a:EGFP* | Not injected | 170 | 11% | 100% normal | 100% normal |
|  | mMO 50 μM | 155 | 14% | 100% normal | 100% normal |
|  | hMO 50 μM | 230 | 98% | Lethality during gastrulation | n.e. |
| *Notch:mCherry* | Not injected | 60 | 0% | 100% normal | 100% normal |
|  | mMO 10 μM | 39 | 3.5% | 100% normal | 100% normal |
|  | hMO 10 μM | 44 | 62% | Delayed, malformed | Delayed but layered |
| *ptf1a:EGFP* | Not injected | 48 | 24% | 100% normal | 100% normal |
|  | mMO 10 μM | 160 | 39% | 100% normal | 100% normal |
|  | hMO 10 μM | 203 | 49% | Delayed, malformed | Delayed but layered |
| *pax6b:GFP / ptf1a:DsRed* | Not injected | 7 | 0% | 100% normal | 100% normal |
|  | mMO 10 μM | 45 | 2.5% | 100% normal | 100% normal |
|  | hMO 10 μM | 59 | 75% | Delayed, malformed | Delayed but layered |
| *neurod1:GFP / ptf1a:DsRed* | Not injected | 50 | 20% | 100% normal | 100% normal |
|  | mMO 10 μM | 110 | 35% | 100% normal | 100% normal |
|  | hMO 10 μM | 150 | 78% | Delayed, malformed | Delayed but layered |

**Supplementary Table S5**

**Rescue experiments in zebrafish**

The table includes raw data on mortality and phenotype of zebrafish embryos injected with control, morpholino, mRNA and rescuing solutions. Data derive from three independent experiments and correspond to the chart of Supplementary Fig. S1.

| **Condition** | **# embryos** | **Mortality**  **at 48 hpf** | **Phenotype of survivors**  **at 48 hpf** |
| --- | --- | --- | --- |
| Not injected | 135 | 0 % | Normal |
| mismMO 10 μM +  C3H mRNA 25 ng/µl | 136 | 3 % | Normal |
| AEY69 mRNA  25 ng/µl | 60 | 23 % | Delayed and/or  malformed (cyclopia) |
| histMO 10 μM | 146 | 50 % | Blocked at somitogenesis |
| histMO 10 μM +  AEY69 mRNA 25 ng/µl | 129 | 57 % | Blocked at somitogenesis, malformed |
| histMO 10 μM +  C3H mRNA 25 ng/µl | 95 | 8 % | 84% normal,  16% delayed |
| histMO 10 μM +  C3H mRNA 50 ng/µl | 77 | 24 % | 75% normal,  25% delayed |

**Supplementary Figures:**

**Fig. S1**

**
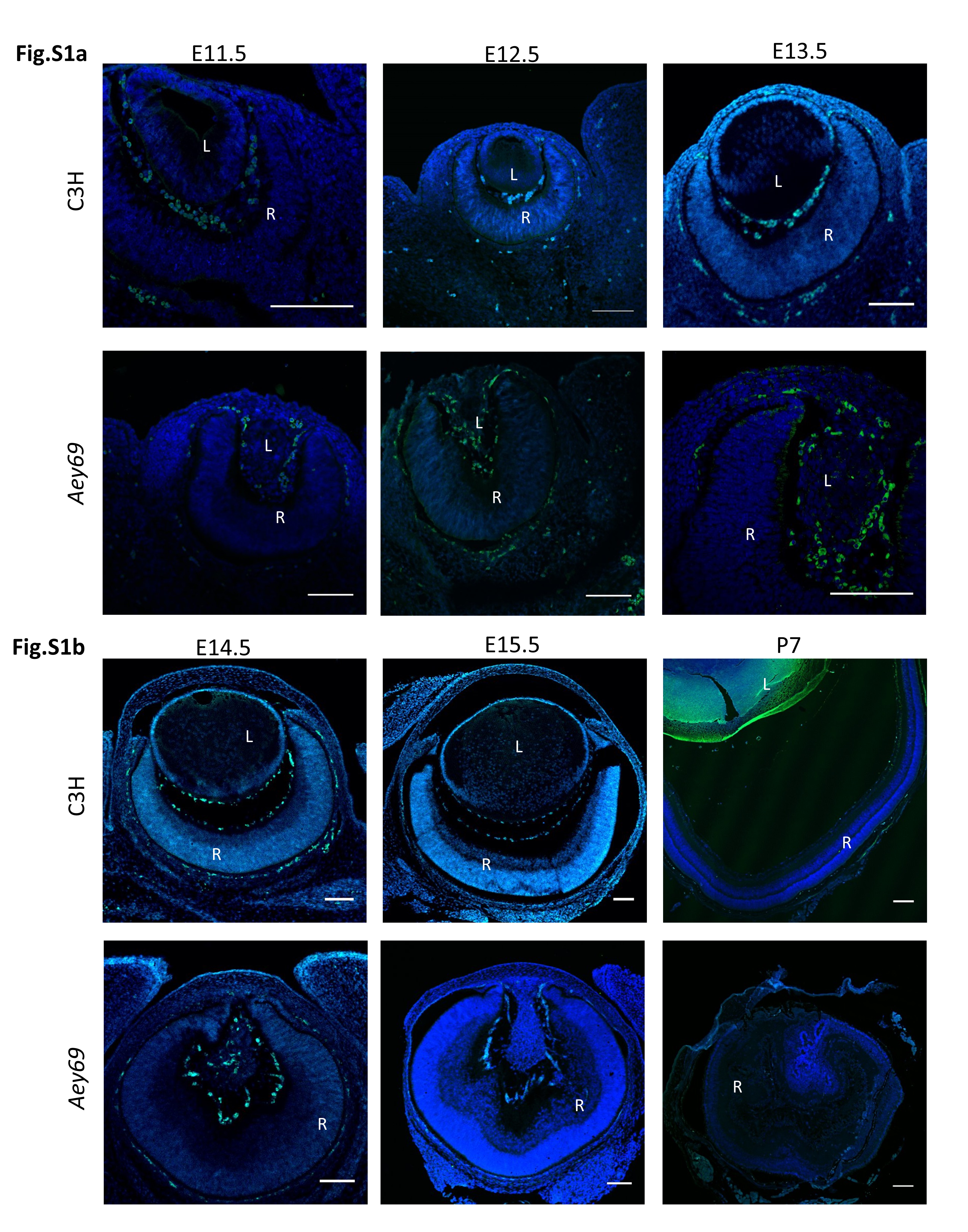

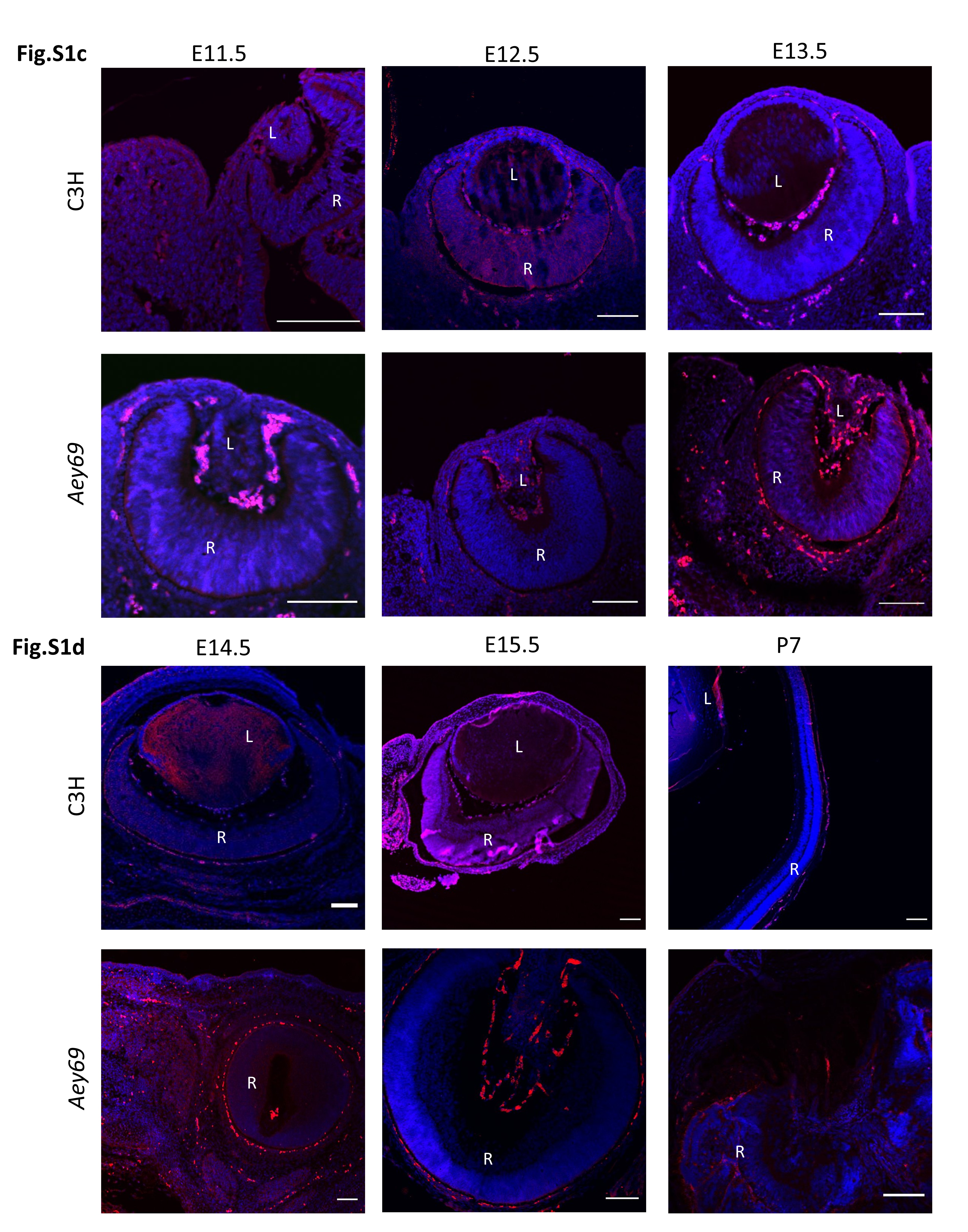
**

Fig. S1: **Representative negative controls**

The negative controls represent the staining without the addition of any primary antibody. This could help in identification of any possible autofluorescent area (e.g. RPE, disrupted mesodermal cells and blood vessels). Any conclusion from the antibody staining was made in comparison to the respective negative control (a: E11.5-E13.5; b: E14.5-P7); no analysis was done in regions showing autofluorescence. DAPI (blue) was used for counterstaining. The bars indicate 100 µm. L, lens; R, retina. Fig. S1a and b represent the autofluorescent areas stained by the secondary A488 antibody, and Fig. S1c and d represent the autofluorescent areas stained by the Cy3 antibody.

**Fig. S2**

**
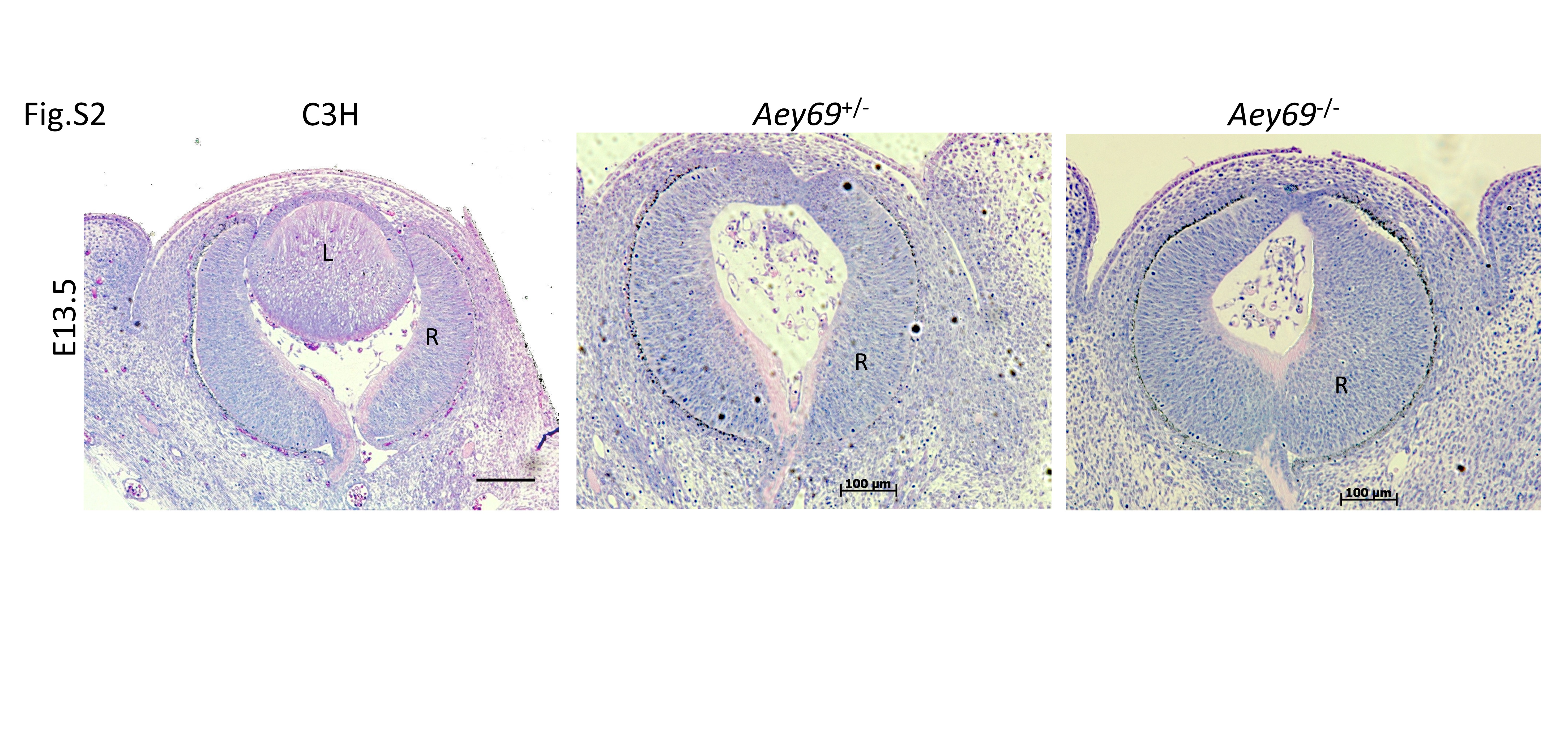
**

Fig.S2: **Histological characterization of ocular defects in wild type, heterozygous and homozygous mutant eye**

The comparative histological staining between the wild type, heterozygous and homozygous mutant eye at E13.5 is shown. No lens is present neither in heterozygous nor in homozygous mutant eyes. Bars indicate 100 µm; L, lens; R, retina; ON, optic nerve.

**Fig. S3**

**
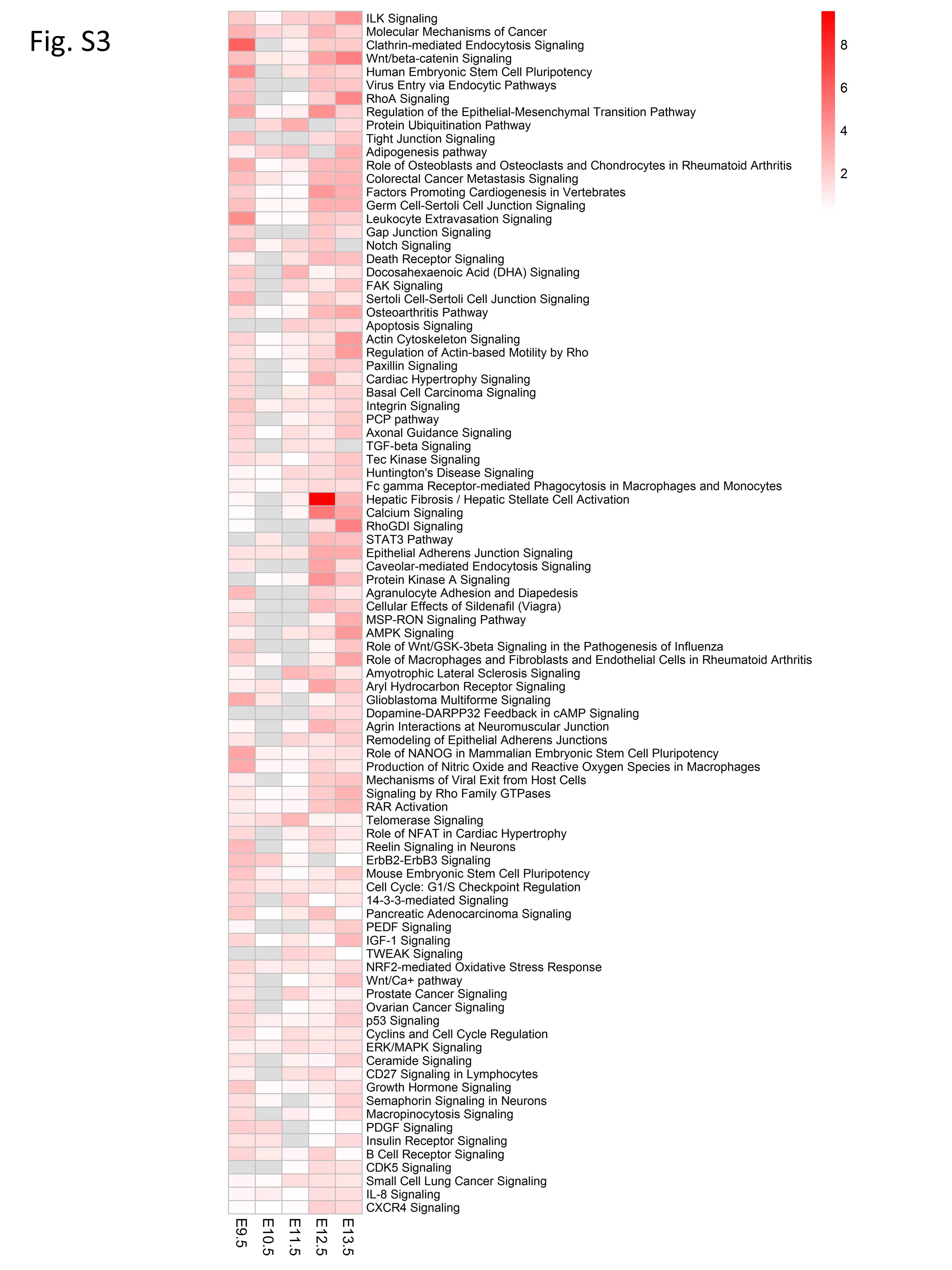
**

Fig. S3. **Heatmap with enrichment p-values from the Ingenuity Pathway Analysis software for canonical pathways**

Grey boxes indicate instances where no p-value was calculated. Pathways shown are ordered by the number of embryonic stages where they are significantly enriched (at least two stages) and by the average of -log(p-value).

**Fig. S4**


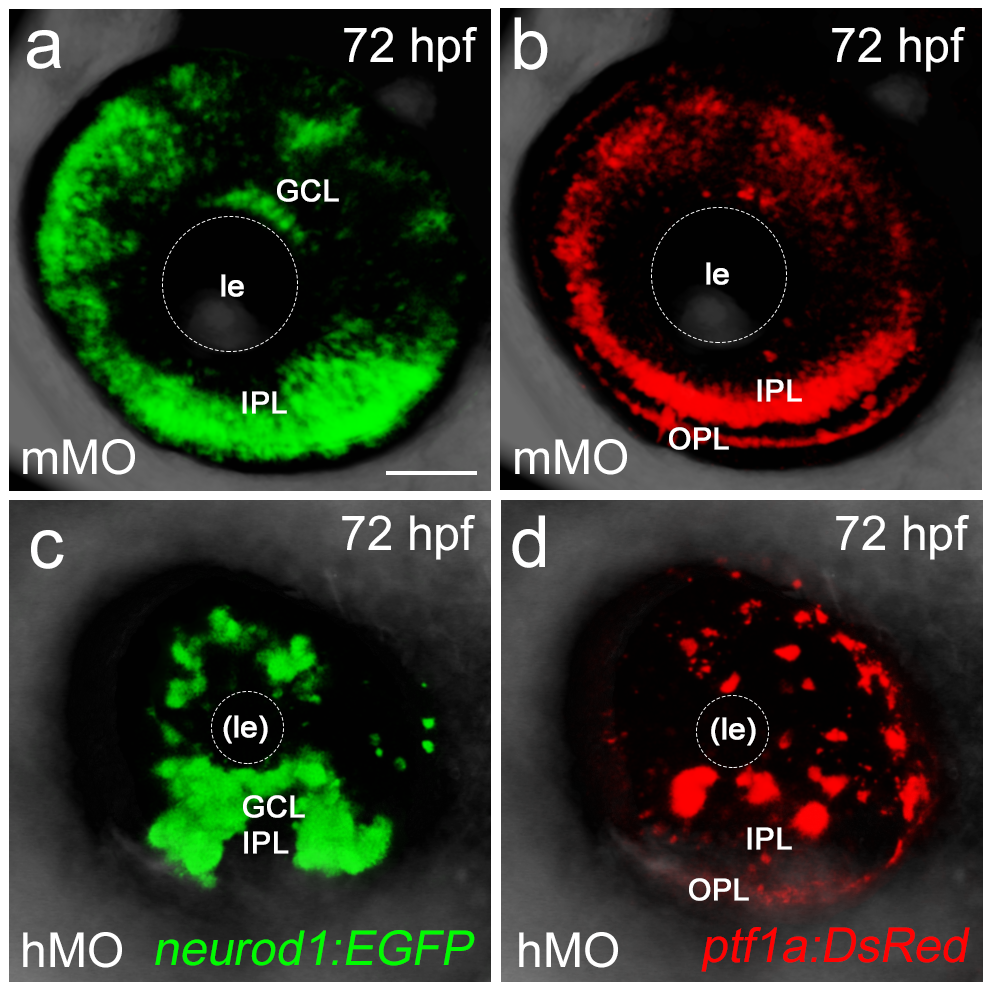


Fig. S4: **Delayed but still layered retina upon knock-down of zebrafish *hist2h3ca1***

a-c: After MO-mediated knockdown of zebrafish *hist2h3ca1* in a double *ptf1a/neurod1* transgenic line, zebrafish morphant embryos (hMO) display a strongly delayed but still layered retina (c,d), compared to controls (mMO) (a,b). *neurod1*-positive cells are specifically detectable in the inner retina (c), occupying the areas of the prospective ganglion cell (GCL) and inner plexiform (IPL) layer, while *ptf1a*-positive cells are arranged in both inner and outer retina (d), in the prospective IPL and outer plexiform layer (OPL). All panels display lateral views of zebrafish eyes at 72 hpf (hours post-fertilization), with anterior to the left. Displayed phenotypes are representative of n=50 embryos per condition. The scale bar (in a) is 50 µm and applies to all images.

**Fig. S5**


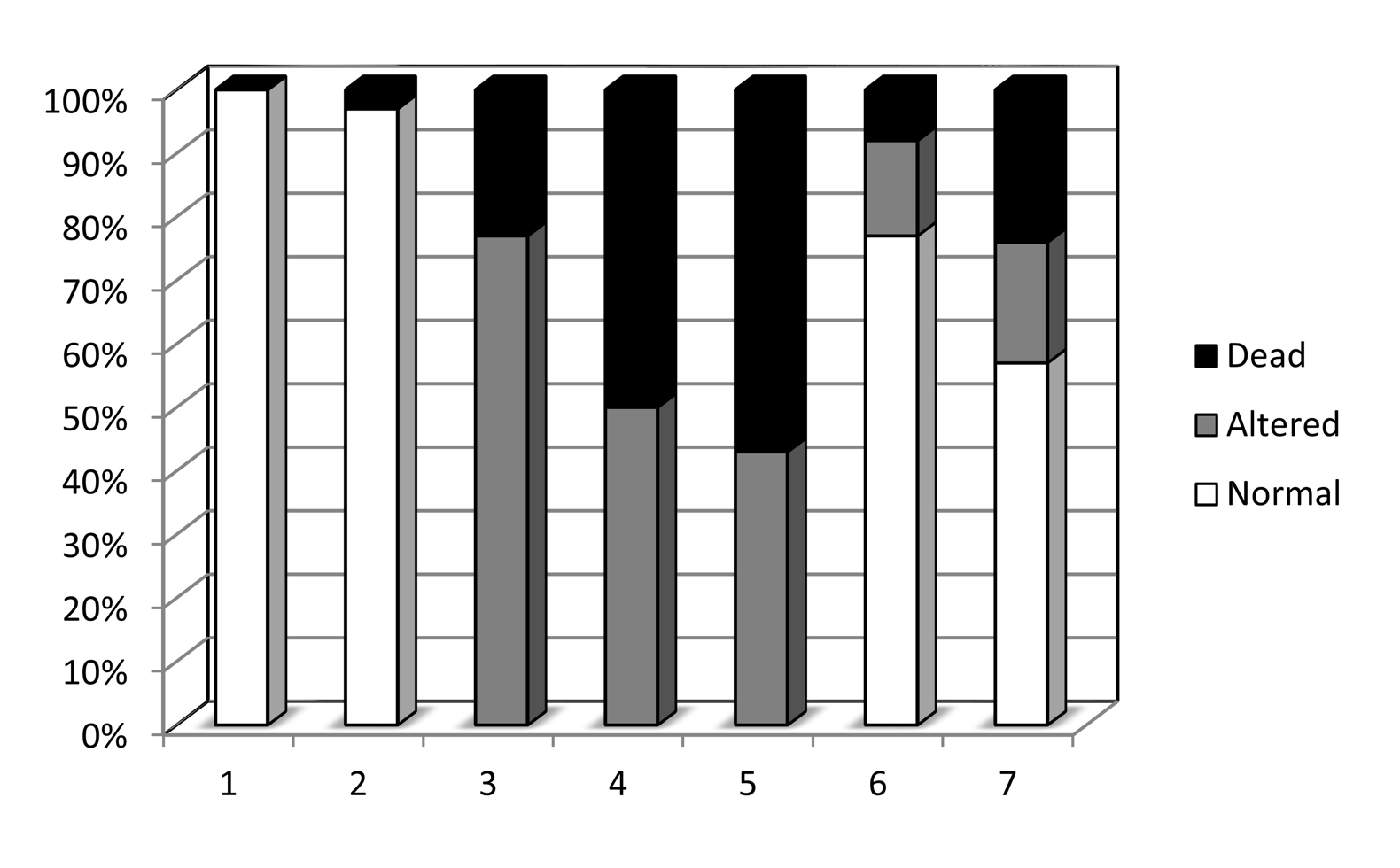


Fig. S5: **Rescue experiments in zebrafish embryos**

The chart summarizes the results from rescue experiments using control (mismMO) or anti-*hist2h3ca1* (histMO) morpholino oligos, injected either alone or in combination with normal (C3H) or mutated (AEY69) mouse mRNAs. Injected controls (2) do not significantly differ from not injected embryos (1). The morphant (histMO) rescue is obtained using normal C3H mRNA (6, 7), while the mutated mRNA is exacerbating the phenotype (5) compared to AEY69 or histMO alone (3, 4). The rescue is more efficient with C3H mRNA at 25 ng/μl (6) than at 50 ng/μl (7). Altered phenotypes include delayed development, malformation and cyclopia. Phenotypes are evaluated at 2 dpf. Conditions: 1) Not injected; 2) mismMO 10 μM + C3H mRNA 25 ng/μl (injected controls); 3) AEY69 mRNA 25 ng/μl (over-expressed); 4) histMO 10 μM (morphants); 5) histMO 10 μM + AEY69 mRNA 25 ng/μl (no rescue); 6) histMO 10 μM + C3H mRNA 25 ng/μl (>70% rescue); 7) histMO 10 μM + C3H mRNA 50 ng/μl (>50% rescue). Sample size: n=778 (raw data available in Suppl. Tab. S5.

Analysis of TH-positive cells in the brain of the *Aey69* mice.

Mice were perfused with phosphate buffered saline (PBS), followed by 4% paraformaldehyde (PFA) in PBS and incubated at 4°C overnight. The brains were transferred into 25% sucrose in PBS for cyroprotection and horizontally cut with a cryostat at 40 µm. Slides were washed in PBS over-night and incubated in 0.1% H_2_O_2_ in PBS to reduce background signal by endogenous peroxidases. 2% FCS in PBS containing 0.1% Triton was used for blocking. For staining, an anti-TH antibody from Pel-Freez (1:1000) was used visualized by a DAB peroxidise-based detection system. Dopaminergic and noradrenergic neurons were quantified by unbiased stereology using the optical fractionator method and analyzed with the Stereoinvestigator software (MicroBrightField Inc., USA). The innervation into the striatum was analyzed using the ImageJ program (Schneider et al., 2012).

Reference:

Schneider, C.A., Rasband, W.S., Eliceiri, K.W., 2012. NIH Image to ImageJ: 25 years of image analysis. Nature Methods 9, 671-675.


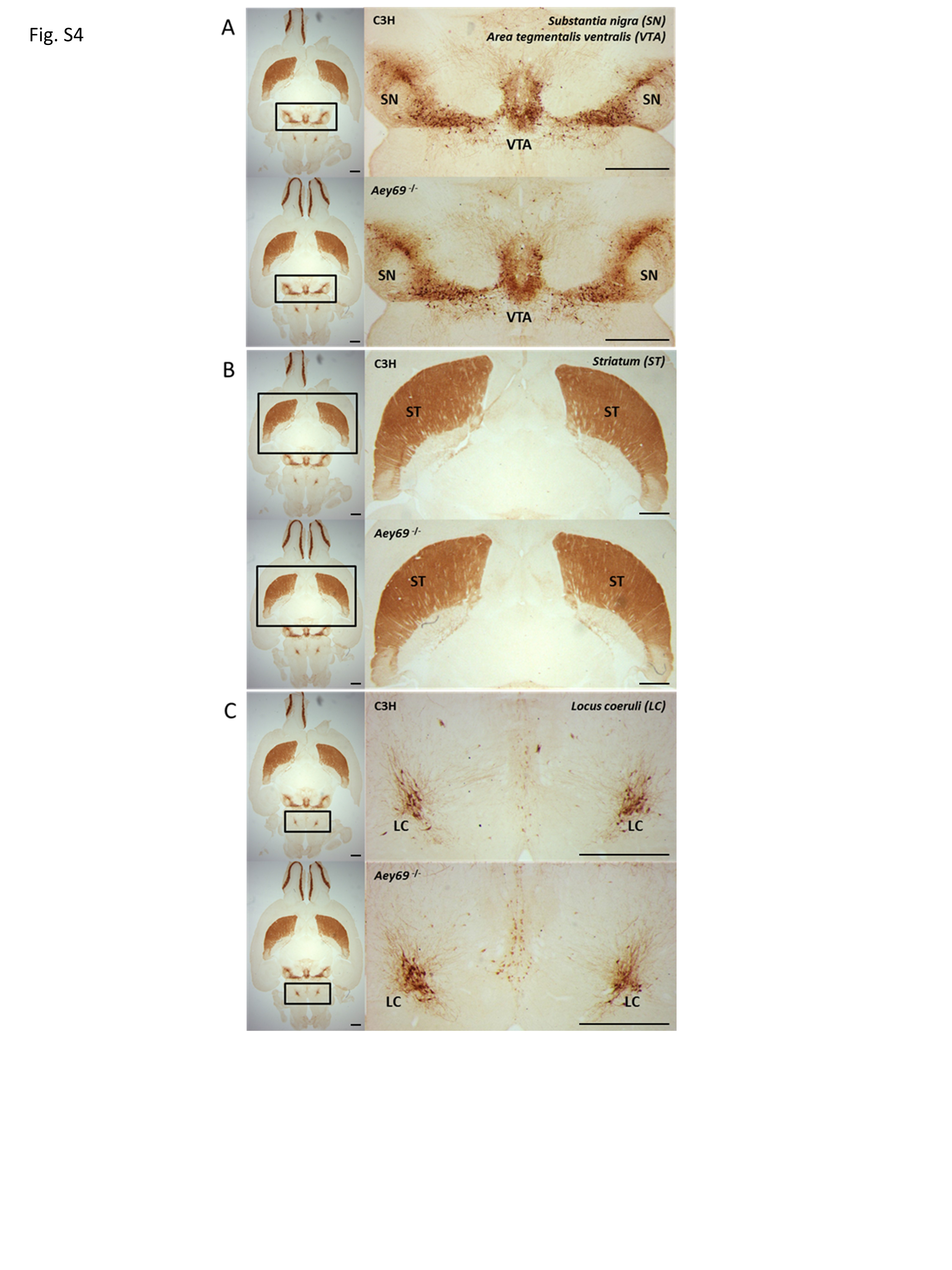


Fig. S6: **The *Aey96* mutation does not affect tyrosine hydroxylase (TH) expression in the adult mouse brain**

The DAB staining represents immunohistological label of tyrosine hydroxylase (TH) positive cells in the brain of the wild-type and the mutant mouse at the age of 9-10 weeks. (a) Stereological counting did not show significant differences in the number of TH-positive cells in the *substantia nigra* (SN) (wild type 10960±766 counts, mutant 10248±1170 counts) and the *area tegmentalis ventralis* (VTA) (wild type 15968±1455 counts, mutant 15257±616 counts). (b) The intensity of the innervation of the dopaminergic neurons from the SN into the striatum (ST) did no differ between the groups. (c) TH-labeled noradrenergic neurons showed no quantitative difference in the mutant brains compared to the wild-type brains (wild type 3738±334 counts, mutant 3720±472 counts; n=6); scale bar = 1 mm.
